# Exosome mediated delivery of Epigallocatechin 3 gallate as a novel approach to alleviate psoriasis symptoms through cytokine and apoptotic pathway modulation

**DOI:** 10.1038/s41598-025-10886-2

**Published:** 2025-08-16

**Authors:** Mohamed S. Kishta, Ahmed A. Abd-Rabou, Garo K. Sarkissian, Ahmed I. Elwakil, Dana M. Elsabry, Youssef M. Zagzoug, Sohaila R. Hussein, Ahmed N. Abdallah

**Affiliations:** 1 Hormones department, Medical Research and clinical studies institute, National Research Centre, Dokki, Cairo, 12622 Egypt; 2 Stem Cell Lab, Center of Excellence for Advanced Sciences, National Research Centre, Dokki, Cairo, 12622 Egypt; 3 Biotechnology Faculty, October University for Modern Sciences and Arts (MSA), 6th of October City, Giza, 12451 Egypt; 4 Researcher of Biotechnology, Hormones Department, Medical Research and Clinical Studies Institute, National Research Centre, Giza, 12622 Egypt

**Keywords:** Psoriasis, Bone marrow mesenchymal stem cells, Stem cell-derived exosomes, Epigallocatechin-3-gallate nanoparticles, Epigallocatechin-3-gallate-loaded exosomes, Nanobiotechnology, Regenerative medicine, Stem-cell biotechnology, Mesenchymal stem cells

## Abstract

Psoriasis is a chronic skin disorder with significant individual and societal impacts. Current therapies often lack efficacy, are costly, or cause side effects, necessitating new treatments. This study explores regenerative therapies—exosomes, mesenchymal stem cells (MSCs), epigallocatechin-3-gallate nanoparticles (EGN), and EGN-loaded exosomes (EGN-Exo)—in regulating psoriasis-related markers (IL-6, IL-4, Bcl-2, Bax, NF-κB, CDC25B). An imiquimod-induced psoriasis model in Wistar rats was used, with six groups: negative control, positive control, and treatments (MSCs, exosomes, EGN, EGN-Exo). After seven days, ELISA revealed EGN-Exo most effectively reduced pro-inflammatory IL-6 and pro-apoptotic Bax while increasing anti-inflammatory IL-4 and anti-apoptotic Bcl-2. EGN-Exo also significantly lowered NF-κB and CDC25B, demonstrating superior anti-inflammatory effects. Apoptosis profiling showed EGN-Exo reduced late apoptotic cells, highlighting cytoprotective abilities. EGN had a moderate effect, while MSCs and exosomes showed modest improvements. Histopathological and immunohistochemical analyses confirmed EGN-Exo’s efficacy, notably reducing TGF-β expression. These findings suggest EGN-Exo combines EGCG’s antioxidant and anti-inflammatory properties with exosomes’ targeted delivery, offering a promising advanced therapy for psoriasis.

**Supplementary Information:**

The online version contains supplementary material available at 10.1038/s41598-025-10886-2.

## Introduction

A chronic inflammatory skin condition mediated by the immune system, psoriasis is linked to several morbidities, such as psoriatic arthropathy, cardiovascular, psychiatric, and hepatic disorders. Skin functions as a neuroimmuno-endocrine organ, capable of sensing environmental stressors and producing hormones and neurotransmitters that influence local and systemic responses. These skin-derived signals may indirectly affect melanocyte stem cell behaviour and hair pigmentation through stress-related pathways^1^. In 2014 WHO classified psoriasis as an epidemic non-communicable disease and noted the distress triggered by inaccurate diagnosis, inadequate treatment, and discrimination against the disease. Psoriasis affects both females as well as males, however, it generally appears earlier in females, along with those with a family background of the disease. The bimodal distribution of its beginning age shows that it increases 10 years earlier in females and at 30–39 and 60–69 years in males^2^. Statistics documented that there are over 1 million Egyptian patients had psoriasis, of which 145,000 are characterized by moderate as well as severe cases^3^.

**Fig. 1:**
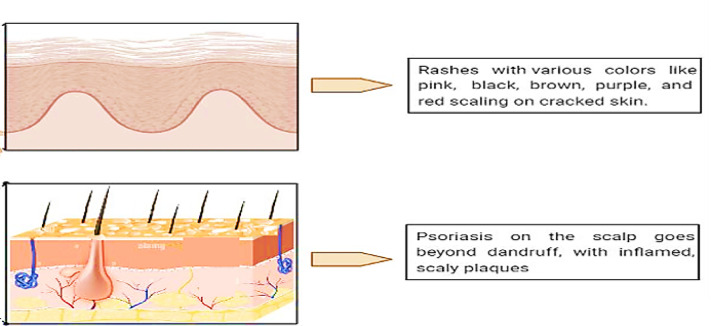
Psoriasis can appear as dandruff-like scaling or widespread rashes with colors ranging from pink to brown, often with red scaling on cracked skin.

Psoriasis is characterized by the extent of skin involvement and intensity of erythema, induration, and scaling as shown in Fig. 1. Psoriatic arthritis, plaque-type psoriasis, pustular psoriasis, inverse psoriasis, guttate psoriasis, nail psoriasis, and erythrodermic psoriasis are the seven main forms of psoriasis^2^. Psoriasis can be successfully managed utilizing several kinds of drugs that focus on certain aspects based on the severity of the condition and specific circumstances of every patient. Many of these treatments have serious side effects, even though they can effectively control symptoms. These include calcineurin inhibitors (which cause itching and stinging), glucocorticoid steroids (which cause skin atrophy after prolonged use), retinoid (which causes redness, peeling, dryness, itching, and burning), and psoralens plus UVA exposure (which increases the risk of skin cancer)^4^. Meanwhile, after new techniques and methods appeared, the most promising effective treatments were stem cells, specifically mesenchymal stem cells (MSCs)^5^. Much research has been done to detect the main effect mesenchymal stem cells on skin diseases, specifically psoriasis which elevates the expression of SOD3 and inhibits the progression as well as severity of psoriasis by regulating immune cell functions and infiltration particularly dendritic cells, Th17 cells, neutrophils and through controlling TLR-7-dependent, MAP kinases, epidermal functions^6^. MSC exosomes can lower the terminal complement complex, C5b-9, and the important psoriatic cytokines, IL-17 and IL-23. Even a minor psoriatic phenotype brought on by three days of IMQ therapy of mouse skin exhibits this^7^. In addition to stem cell-based therapies, major studies have documented the significant effect of epigallocatechin gallate (EGCG) as a treatment for psoriasis. In addition to lowering inflammation and proliferation responses in cultured keratinocytes, EGCG is crucial for differentiation. Among IMQ-induced mouse skin lesions, it produced a notable (*p* < 0.01) decrease in psoriasiform signs and symptoms, such as a decrease in skin and ear thickness, scales, and erythema, as well as a decrease in proliferation (Ki-67), infiltratory immune cells (neutrophils, mast cells, CD4 + T cells, and macrophages), and (CD31) angiogenesis^8^.The complicated inflammatory cascade that causes psoriasis is largely mediated by NF-κB, which intensifies immunological responses and keratinocyte dysfunction. Chronic inflammation is fueled by the transcription of pro-inflammatory cytokines, antimicrobial peptides, and survival proteins that result from NF-κB activation^10^. Furthermore, the CDC25B pathway, which is elevated in psoriatic lesions, exacerbates keratinocyte hyperproliferation by encouraging abnormal cell cycle progression. This cycle is further exacerbated by the dysregulated Th cell subsets, especially Th17 and Th22, which release TNF-α, IL-17, and IL-22, which maintain tissue damage and immune cell infiltration^9^. Prolonged keratinocyte survival and psoriasis persistence are caused by an imbalance between the apoptotic regulators Bcl-2 and Bax. This research seeks to explore the intersection of immunology, regenerative medicine, and clinical innovation in addressing the unmet needs of psoriasis treatment. By delving into the mechanisms and applications of MSCs, exosome therapy, and related technologies, this work aims to highlight a transformative path forward for managing this debilitating condition.

## Results

### Isolation and characterization of stem cells (morphology and flow) and MSC-derived exosomes (TEM and DLS)

**Fig. 2:**
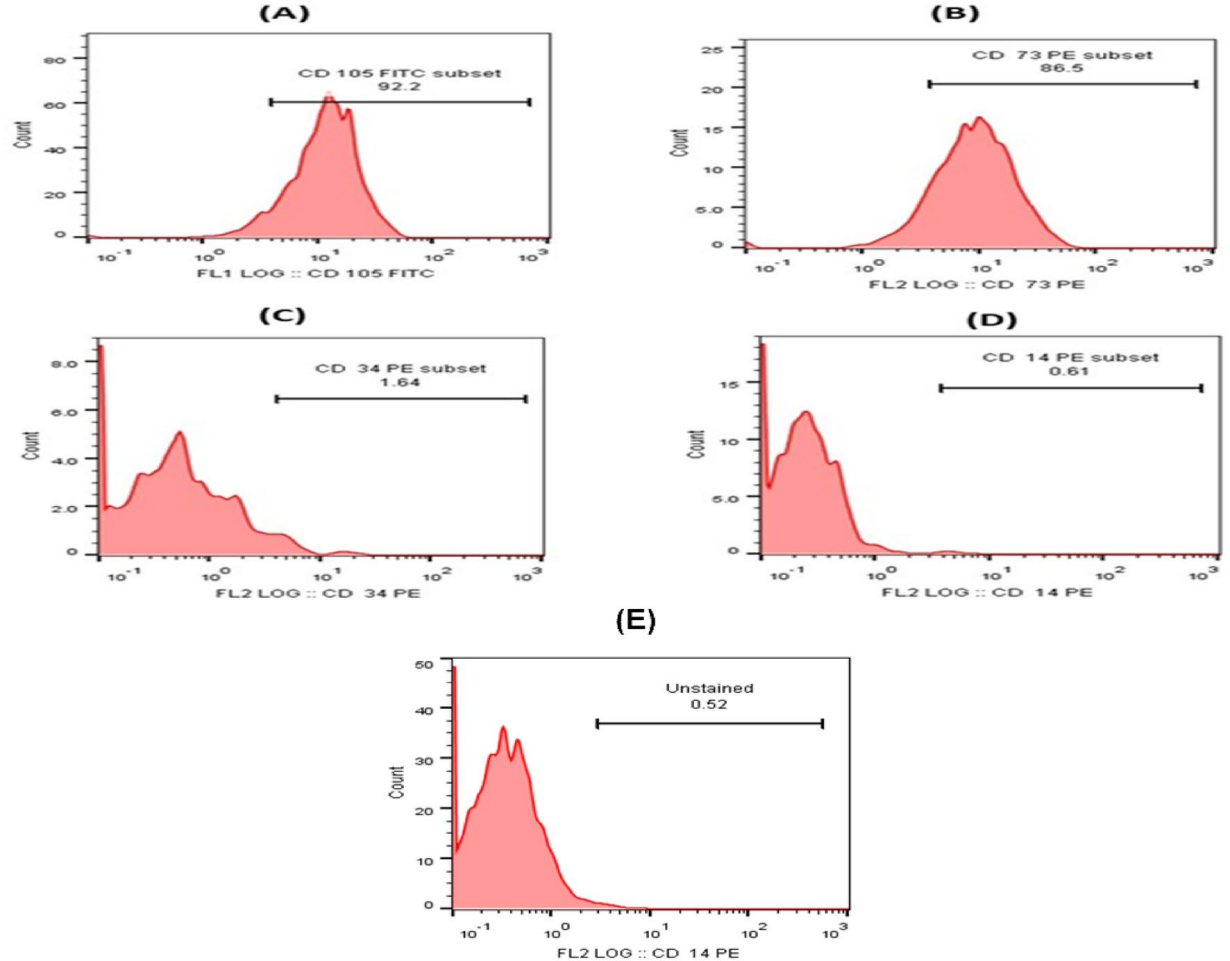
This figure indicates the expression levels of the tested CD markers. (**A**) CD105 is expressed in 92.2%, (**B**) CD73 is expressed in 86.5%, (**C**) CD34 is expressed in 1.64%, and (**D**) CD14 is expressed in 0.61%, (**E**) Unstained cells.

The analysis of the flow cytometry for the characterization of MSCs showed positive expression of the two tested CD markers (CD 105, CD 73), indicating that CD105 is expressed in 92.2% of the sample and CD 73 is expressed in 86.5% of the sample, as shown in Fig. 2. It also showed negative expression of the two tested CD markers (CD34, CD14), as it showed that CD34 is expressed in 1.64% and CD14 is expressed in 0.61%, as shown in Fig. 2 and The complete gating strategy for the flow cytometry experiments is shown in the Supplementary Data S1. The unstained, single-stained, and double-stained samples were retested to ensure clarity and reproducibility.

**Fig. 3:**
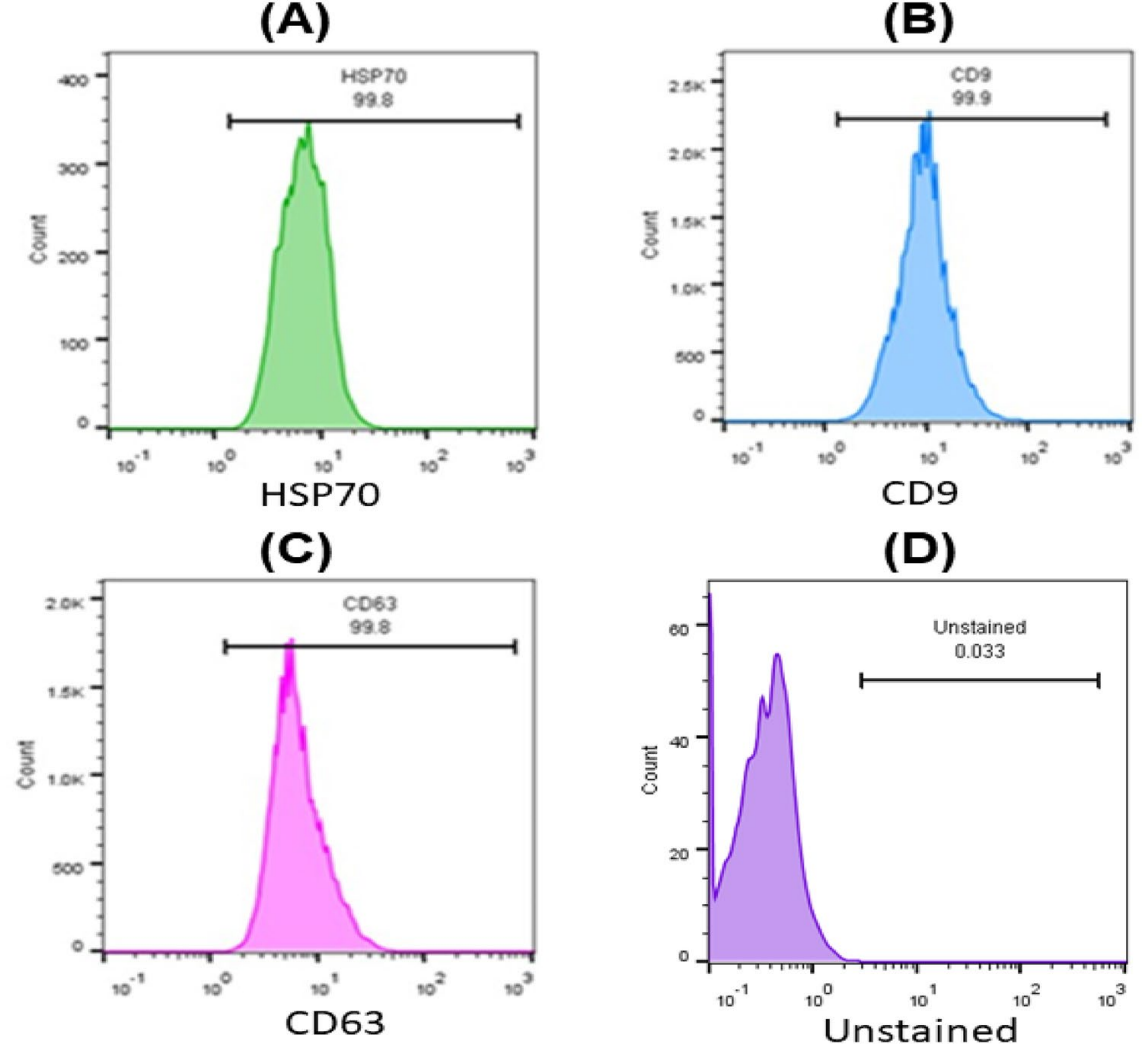
This figure shows the expression levels of CD markers on MSCs-derived exosomes (**A**) HSP70 is expressed in 99.8%, (**B**) CD9 is expressed in 99.9%, and (**C**) CD63 is expressed in 99.8%. (**D**) Unstained MSCs-derived exosomes.

MSCs-derived exosomes were isolated successfully as the analysis of the flow cytometry showed positive expression of the tested markers (HSP70, CD9, and CD63) as it showed that HSP70 is expressed in 99.8% of the sample, CD9 is expressed in 99.9% of the sample, and CD63 is expressed in 99.8% of the sample as shown in Fig. 3.

**Fig. 4:**
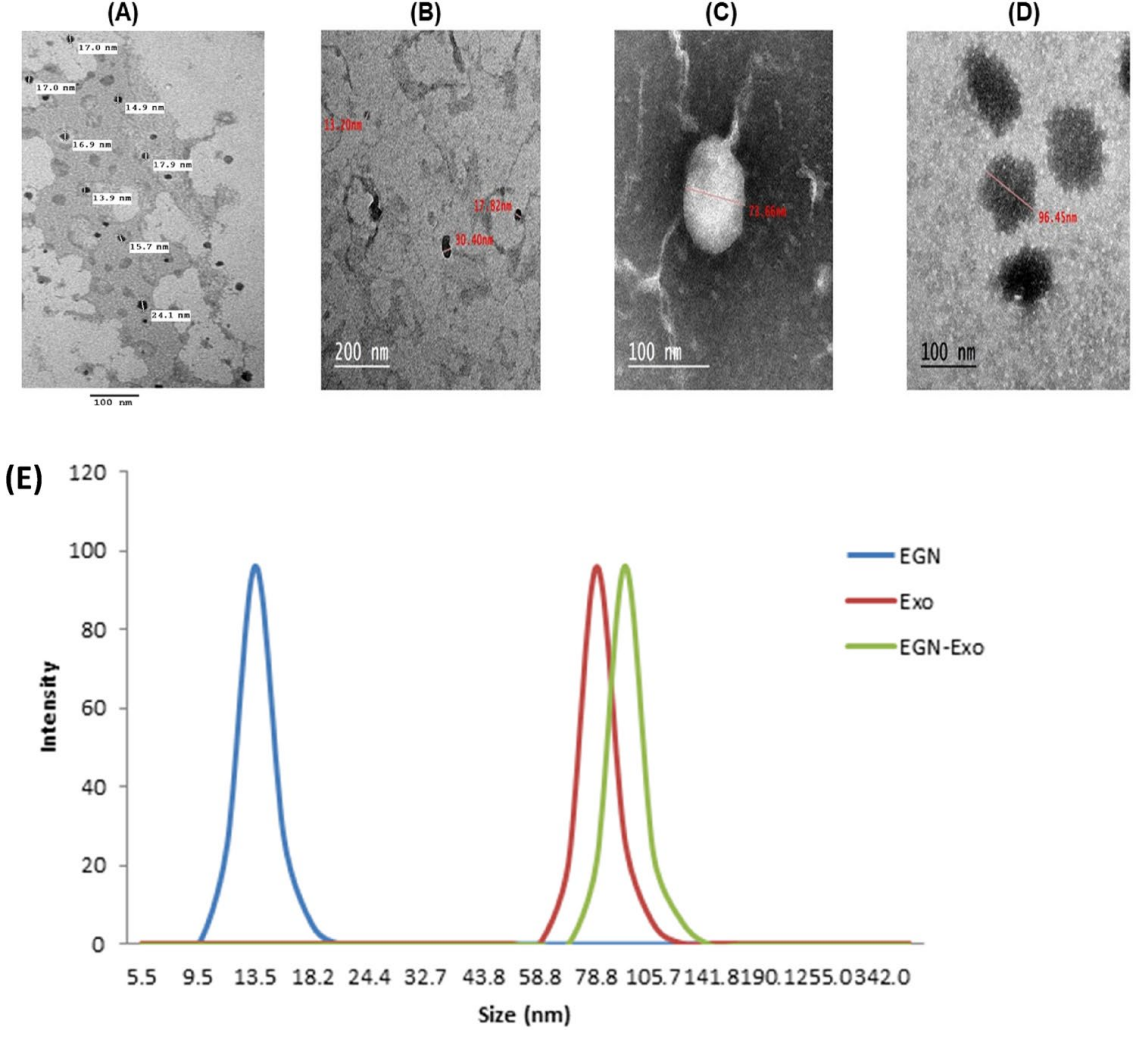
This figure shows the morphological traits of EGCG nanoparticles (**A**, **B**) with an average diameter of 13.5 nm and two magnification powers, Exosomes (**C**) with a diameter of 73.66 nm, and EGCG nanoparticles - loaded exosomes (**D**) with a diameter of 96.54 nm. (**E**) shows the size distribution of EGCG nanoparticles, exosomes, and EGCG nanoparticles-loaded exosomes by Dynamic Light Scattering, and it indicates different sizes of the nanoparticles.

The transmission electron microscopy results showed the morphological traits of nanoparticles, as it showed EGCG nanoparticles with a diameter of 13.5 nm, exosomes with a diameter of 73.66 nm, and EGCG nanoparticle-loaded exosomes with a diameter of 96.54 nm, as shown in Fig. 4.

**Table 1:** Size, polydispersity index, and entrapment efficiency of EGCG nanoparticles, exosomes, and EGCG nanoparticles-loaded exosomes (*n* = 3).

	Size (nm)	PDI	EE%
EGCG Nanoparticles	13.5 *±* 1.11	0.037 *±* 0.00	85.34 *±* 4.30
Exosomes	78.1 *±* 2.31	0.062 *±* 0.02	-
EGCG Nanoparticles- loaded exosomes	96.7 *±* 4.3	0.054 *±* 0.01	73.61 *±* 3.15

Dynamic Light Scattering (DLS) shows the size distribution of nanoparticles, and it showed a uniform peak of EGCG nanoparticles with an average size of 13.5 nm, while Exosomes with an average of 78.1 nm, and EGCG nanoparticles loaded Exosomes with an average of 96.7 nm, as shown in Fig. 4. The size of EGCG nanoparticles, exosomes, loaded exosomes, polydispersity index, and entrapment efficiency were measured, analyzed, and summarized in Table 1.

### In vitro release

**Fig. 5:**
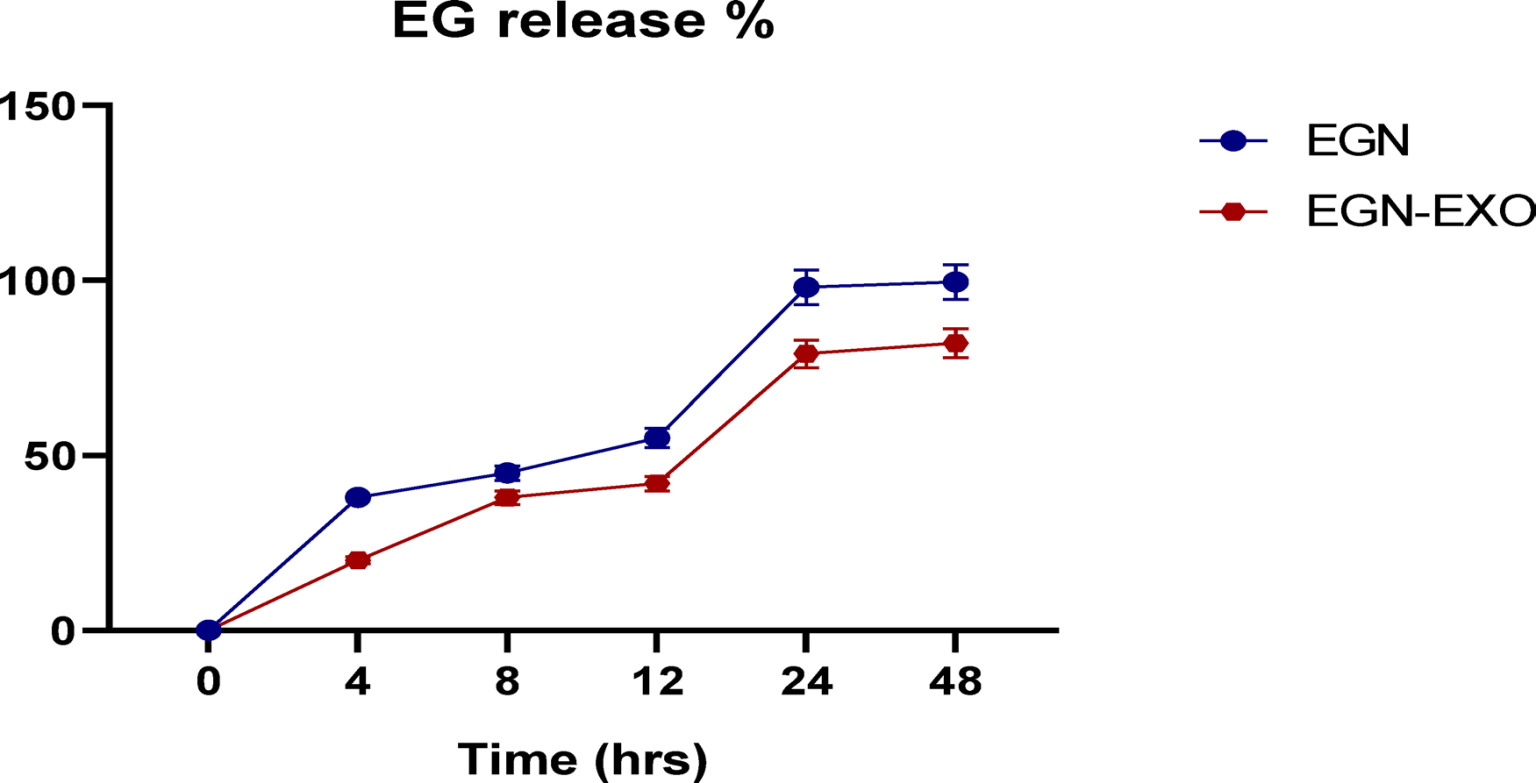
This figure shows that EGCG release was accomplished successfully, with EGCG nanoparticles exhibiting a gradual release of 99.5% over 48 h. The EGCG-loaded Exosomes successfully released 82.1% over 48 h (*n* = 3).

The release of EGCG from EGCG nanoparticles and EGCG nanoparticle-loaded exosomes. The EGCG release was done successfully and indicated that EGCG nanoparticles showed a slow release of 99.5% over 48 h, and EGCG nanoparticles-loaded exosomes also showed a release of 82.1% over 48 h, as shown in Fig. 5.

### Injection of treatment

**Fig. 6:**
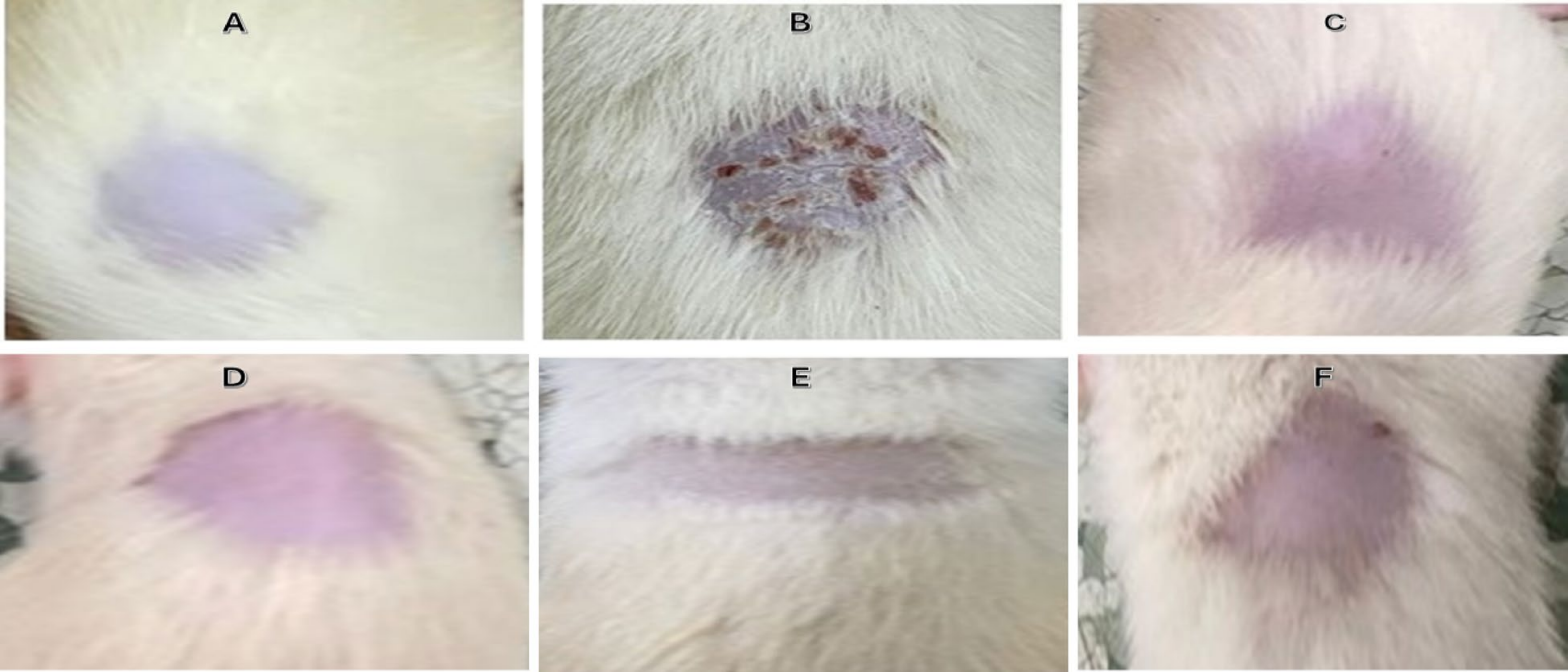
This figure indicates (**A**) the normal rat without induction (negative control), (**B**) the induced rat with psoriasis (Positive control), (**C**) the treated rat with mesenchymal stem cells, (**D**) the treated rat with exosomes, (**E**) the treated rat with EGCG nanoparticles-loaded exosomes and (**F**) the treated rat with EGCG nanoparticles.

The effect of the applied treatments on the induced psoriasis rats in the samples treated with MSCs, MSCs-derived exosomes, EGCG nanoparticles loaded exosomes, and EGCG nanoparticles had great effects on the induced rats, as shown in Fig. 6.

### Biochemical analysis

#### ELISA assay analysis

**Fig. 7:**
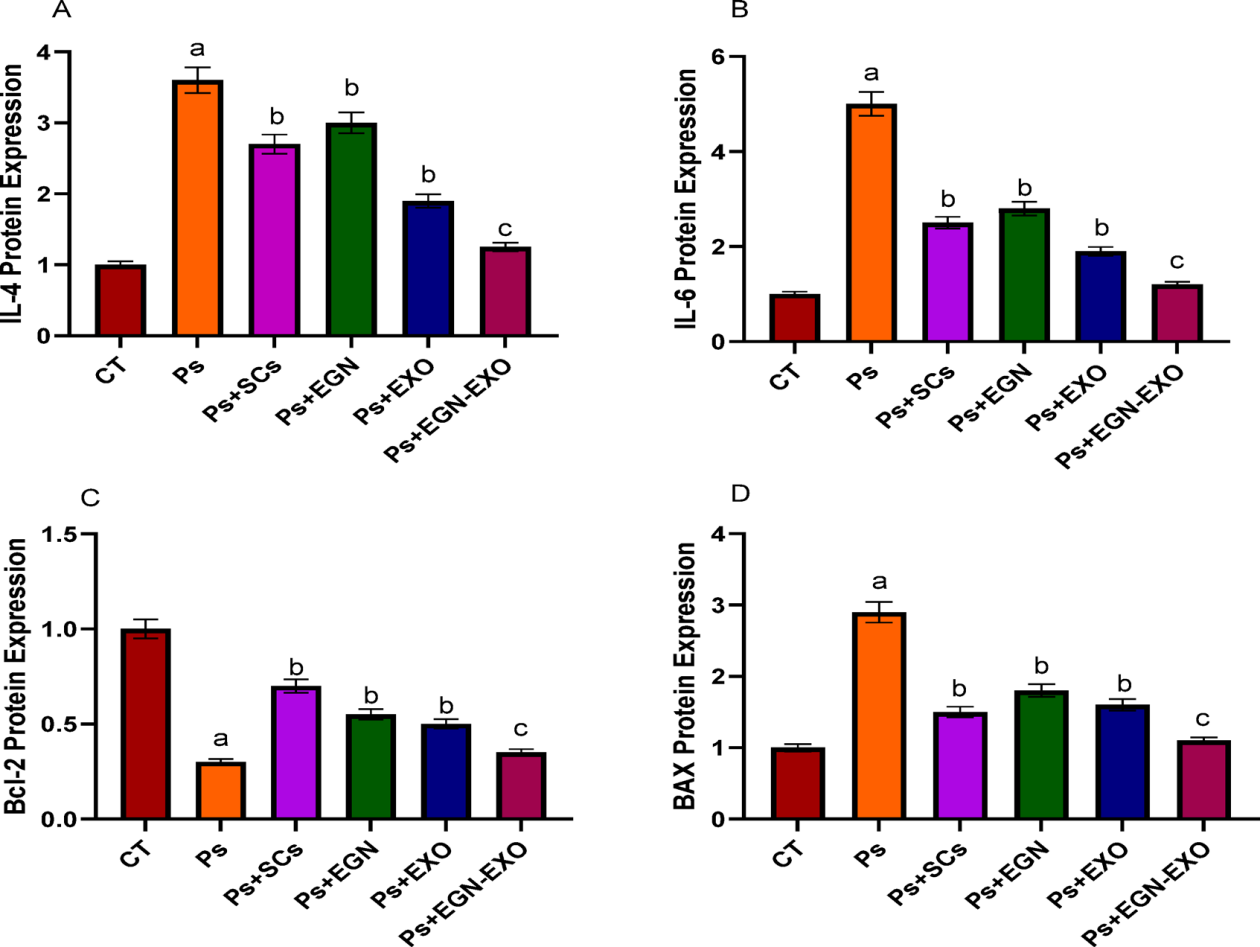
The figure clarifies the level of expression of IL-4 (**A**), IL-6 (**B**), Bcl-2 (anti-apoptotic marker) (**C**), and (pro-apoptotic marker) BAX (**D**) on the negative (CT), positive control (Ps) and treated sample with mesenchymal stem cells (SCs), EGCG Nanoparticles (EGN), Exosomes (Exo) and EGCG Nanoparticles loaded Exosomes (EGN-Exo). The data obtained in the present work were represented in figures and tables as the mean *±* standard error (SE). All assays were repeated three times (*n* = 3). A one-way ANOVA analysis (GraphPad Prism, version 8.0.2) was used. **a**: P values < 0.01 were considered highly statistically significant when comparing the positive control (Ps) with the control, **b**: P values < 0.05 were considered statistically significant when comparing the treated groups (Ps + SCs, Ps + EGN, and Ps + EXO) with the positive control. **c**: P values < 0.01 were considered highly statistically significant when comparing the treated group (Ps + EGN-EXO) with the positive control.

The results of Expression levels of IL-4, IL-6 proteins, as well as apoptosis markers, include Bax and Bcl-2 of ELISA technique indicated the expression of interleukins-4 and − 6 of the control sample, the sample induced with psoriasis, the sample treated with Mesenchymal stem cells, the sample treated with EGCG nanoparticles, the sample treated with exosomes and the sample treated with EGCG nanoparticles- loaded exosomes, in which their level in the psoriasis sample increased more than the normal range while after the treatments were applied their level was decreased as shown in Fig. 7 (A, B). The ELISA results for the expression levels of the Bax (pro-apoptotic marker) and Bcl2 (anti-apoptotic marker) showed that in the psoriasis sample, the Bax level increased to 2.9, which is more than the normal range that is 1, but after the treatments were applied, the level of Bax decreased. While in the case of Bcl-2, its level decreased in the samples induced with psoriasis to 0.3, which is less than the normal range that is 1, but after the treatments were applied, its level increased, as shown in Fig. 7 (C, D).

**Fig. 8:**
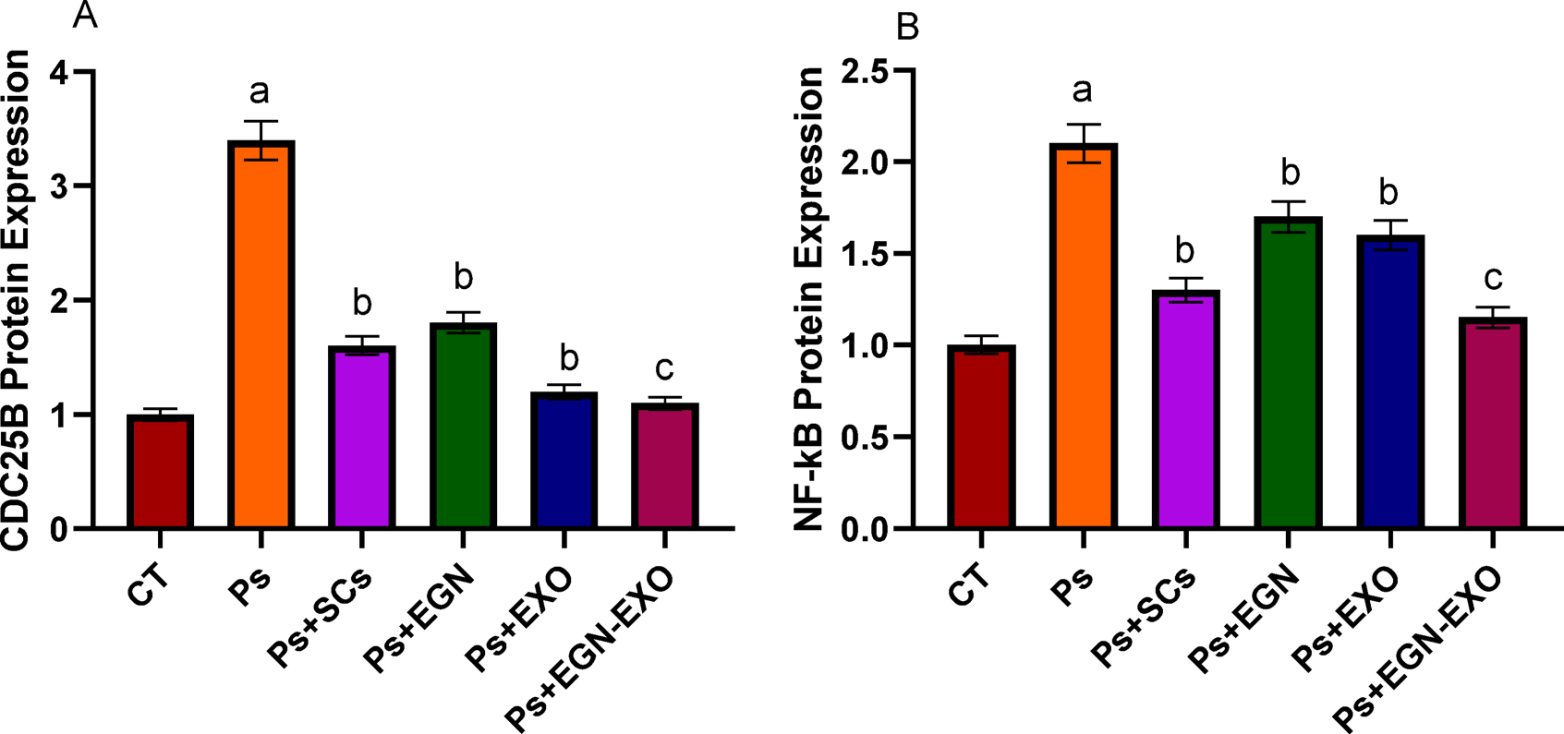
This figure indicates the ELISA results of the expression level of CDC25B (**A**) and NF-kB (**B**) within the negative (CT), positive control (ps) and treated samples with Mesenchymal stem cells (SCs), EGCG Nanoparticles (EGN), Exosomes (Exo) and EGCG Nanoparticles loaded Exosomes (EGN-Exo). The data obtained in the present work were represented in figures and tables as the mean *±* standard error (SE). All the essays were repeated three times (*n* = 3). A one-way ANOVA analysis (GraphPad Prism, version 8.0.2) was used. **a**: P values < 0.01 were considered highly statistically significant when comparing the positive control (Ps) with the control, **b**: P values < 0.05 were considered statistically significant when comparing the treated groups (Ps + SCs, Ps + EGN, and Ps + EXO) with the positive control. **c**: P values < 0.01 were considered highly statistically significant when comparing the treated group (Ps + EGN-EXO) with the positive control.

ELISA results clarified the expression levels of the two pathways, CDC25B and NF-kB, in which their level increased in the case of the psoriasis sample, till CDC25B reached 3.4 and NF-kB reached 2.1, which is more than the normal range, that is 1. After the treatments were applied, the expression levels of the two pathways, CDC25B and NF-kB, decreased to reach near the normal range, as shown in Fig. 8.

#### Complete blood count (CBC)

**Fig. 9:**
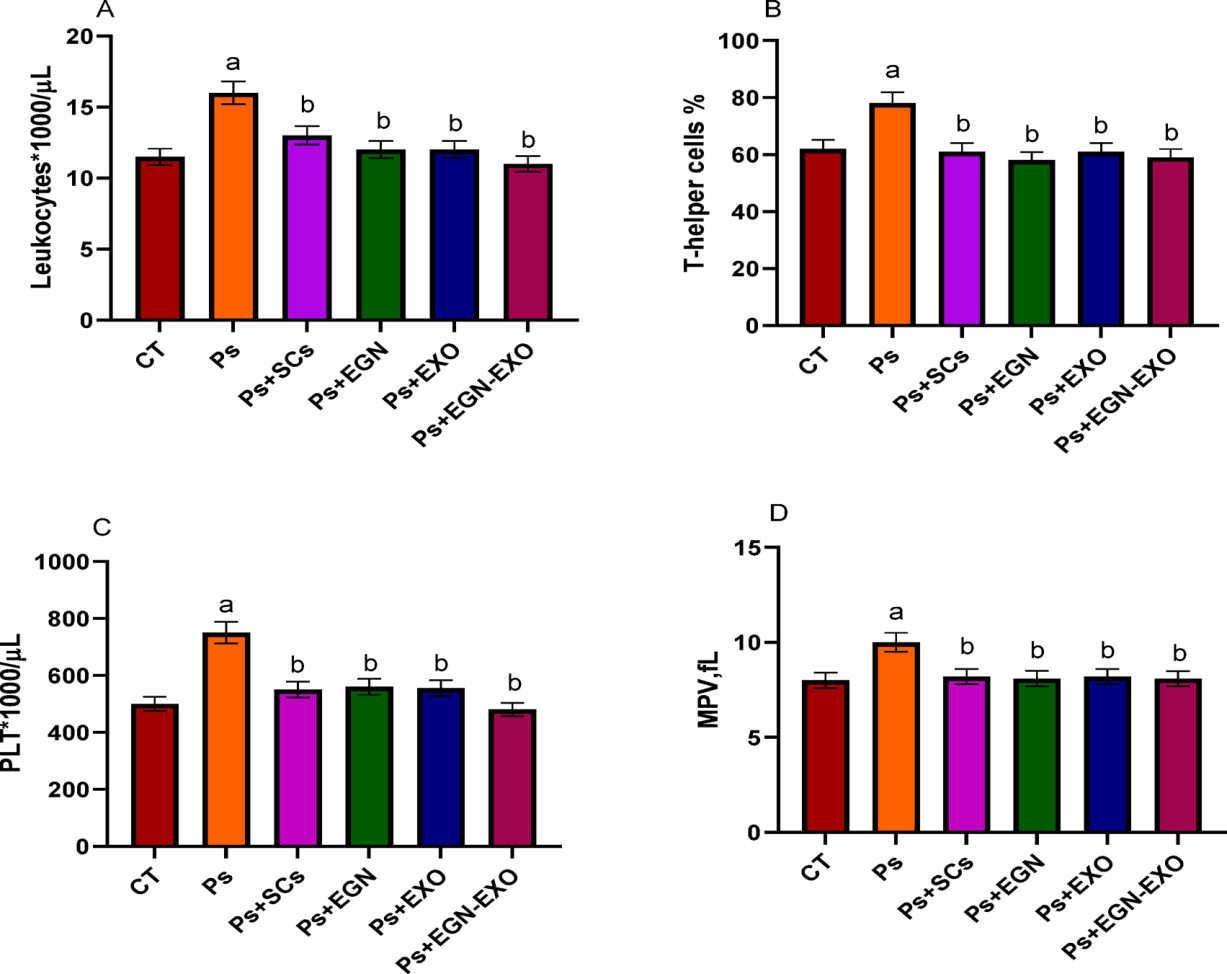
The figure indicates the levels of expression of leukocytes (**A**), T helper cells (**B**), Platelets (**C**) and MPV (**D**) within the negative (CT), positive control (Ps) and treated samples with Mesenchymal stem cells (SCs), EGCG Nanoparticles (EGN), Exosomes (Exo) and EGCG Nanoparticles loaded Exosomes (EGN-Exo). The data obtained in the present work were represented in figures and tables as the mean *±* standard error (SE). All assays were repeated three times (*n* = 3). A one-way ANOVA analysis (GraphPad Prism, version 8.0.2) was used. **a**: P values < 0.01 were considered statistically significant when comparing the positive control with the control. **b**: P values < 0.05 were considered statistically significant when comparing the treated groups with the positive control.

The analysis of CBC indicated that the levels of leukocytes, T helper cells, PLT and MPV are high in the psoriasis sample that for leukocytes is15.9000/ul, for T helper cells is 78%, for PLT is 764,000/ul, and for MPV is 10.1 fl., which is more than the normal range. After the treatments were applied, the levels of leukocytes, T helper cells, PLT, and MPV decreased to reach near the normal level, as mentioned in Fig. 9.

### Flow cytometry (Apoptosis profiling)

**Fig. 10:**
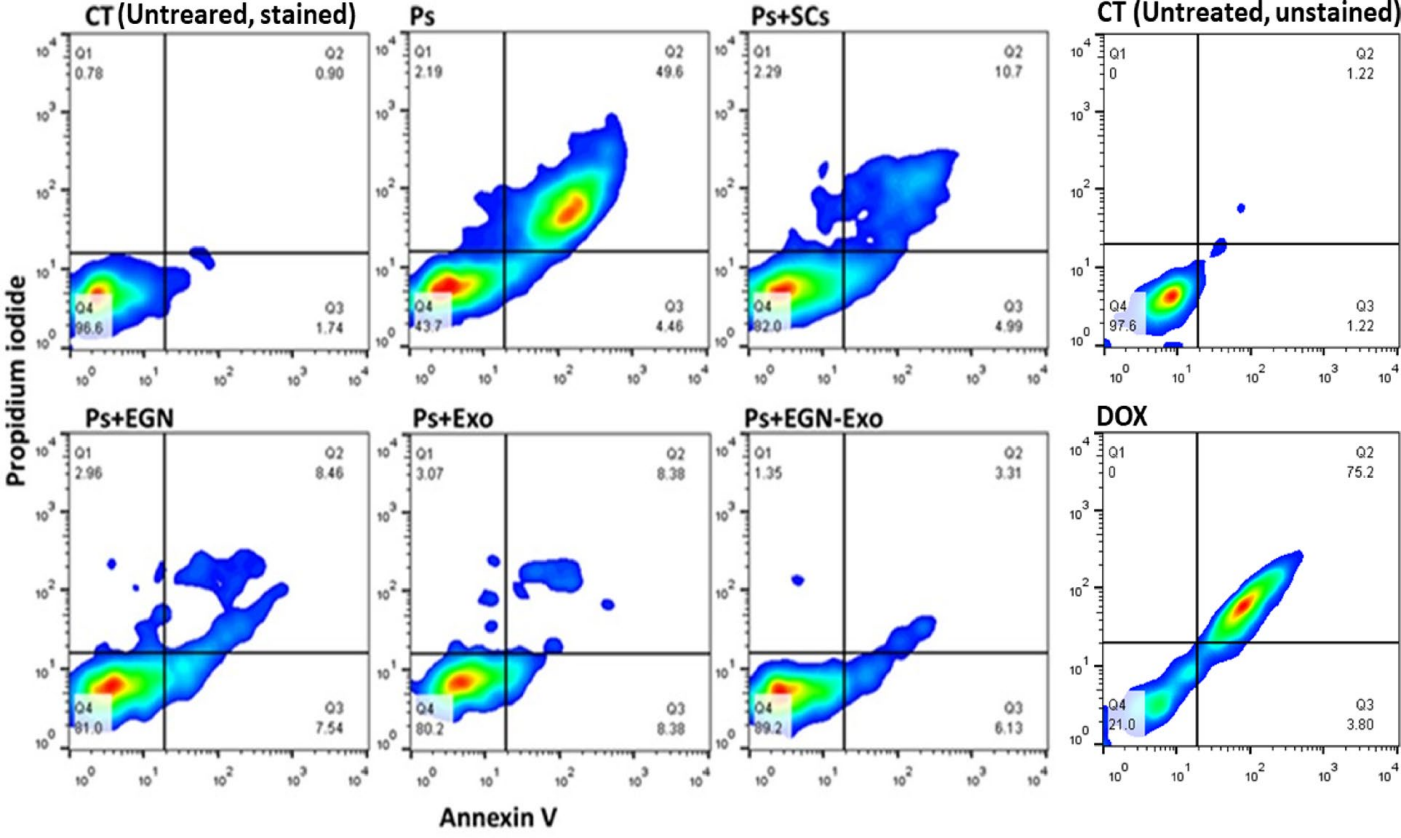
The figure clarifies the apoptosis in the negative control (untreated/unstained cells and untreated/stained cells), positive control (PS-induced and doxorubicin “DOX”-treated cells), and the treated samples, in which Q1 indicates pure necrosis, Q2 indicates late apoptosis, Q3 indicates early apoptosis, and Q4 indicates normal cells.

Apoptosis profiling was conducted using Annexin V and propidium iodide (PI) staining to evaluate the extent of apoptosis among different experimental groups, including the control (CT), psoriasis-induced (Ps), and treated groups: bone marrow-derived mesenchymal stem cells (Ps + SCs), EGCG nanoparticles (Ps + EGN), mesenchymal stem cell-derived exosomes (Ps + Exo), and the combination of both treatments (Ps + EGN-Exo). The results were presented as dot plots with cell populations distributed across four quadrants as shown in Fig. 10: Q1 representing necrotic cells, Q2 late apoptotic cells, Q3 viable cells, and Q4 early apoptotic cells. In the control group, most cells (96.6%) were in Q4 (early apoptosis), with minimal percentages in Q3 (viable, 1.74%), Q2 (late apoptosis, 0.90%), and Q1 (necrosis, 0.78%), reflecting a healthy apoptotic balance. Upon induction of psoriasis, a significant shift was observed, with 49.6% of cells in Q2 (late apoptosis) and 43.7% in Q4 (early apoptosis), indicating extensive cell damage and disrupted homeostasis, while viable and necrotic cells dropped to 4.46% and 2.19%, respectively. Treatment with bone marrow-derived MSCs (Ps + SCs) resulted in a marked improvement, restoring 82.0% of cells to early apoptosis and reducing late apoptotic cells to 10.7%, with slight increases in viability (4.99%) and necrosis (2.29%). The Ps + EGN group exhibited a comparable effect, with 81.0% of cells in early apoptosis, 7.54% viable, and 8.46% in late apoptosis, suggesting effective anti-inflammatory and cell-regulatory properties of EGCG nanoparticles. Similarly, Ps + Exo treatment yielded 80.2% early apoptotic cells, 8.38% viable, and 8.38% in late apoptosis, further supporting the immunomodulatory role of MSC-derived exosomes. Notably, the combination treatment (Ps + EGN-Exo) demonstrated the most promising outcome, with the highest percentage of early apoptotic cells (89.2%), reduced late apoptosis (3.31%) and necrosis (1.35%), and enhanced cell viability (6.13%), highlighting a synergistic effect that offers enhanced protection and restoration of cellular equilibrium in psoriatic conditions.

### Pathological assay

**Fig. 11:**
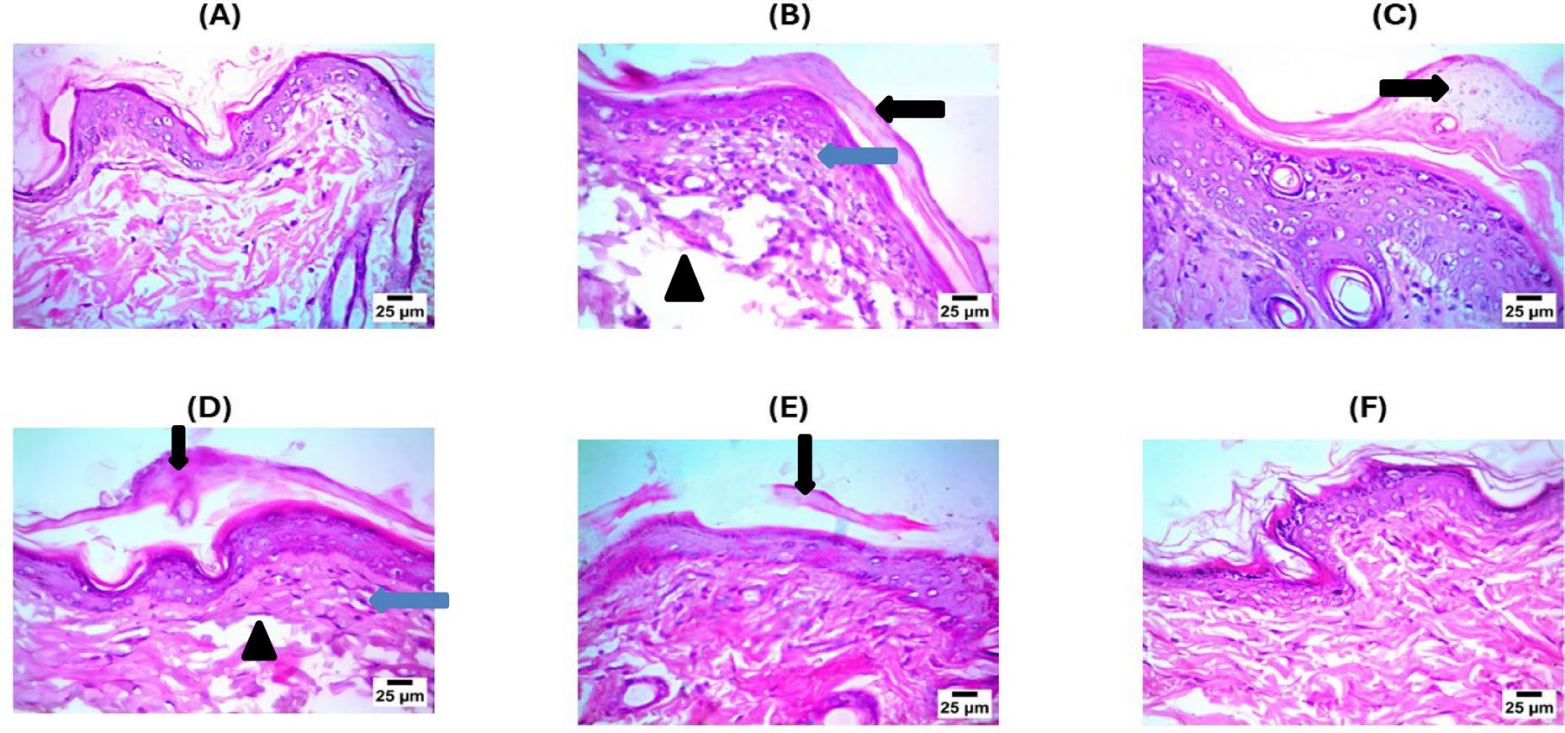
This figure indicates normal histological structure in the negative control (**A**), showing parakeratosis (black arrow), infiltration of dermis by high number of mononuclear inflammatory cells (blue arrow) with sever edema(arrow head) in the untreated samples (positive control) (**B**), showing parakeratosis (black arrow) with normal histological structure of epidermis and dermis in the treated group with Mesenchymal stem cells (**C**), showing parakeratosis (black arrow), infiltration of dermis by few number of mononuclear inflammatory cells (blue arrow) with mild edema (arrow head) in the sample treated with EGCG Nanoparticles (**D**). While a mild parakeratosis (black arrow) with mild infiltration with mononuclear inflammatory cells was observed in the samples treated with exosomes (**E**). The photomicrograph of the treated group, EGCG Nanoparticles loaded into Exosomes (**F**), shows normal histological structure of dermis and epidermis.

The histopathology results showed abnormal histological structure in the untreated psoriasis samples, as shown in Fig. 11, as the results showed the presence of parakeratosis and infiltration of the dermis by a huge number of mononuclear inflammatory cells, and with severe edema in the untreated samples. In the case of the treated group with MSCs photomicrograph illustrates parakeratosis (with normal histological structure of epidermis as well as dermis). The samples treated with EGCG nanoparticles displayed mild parakeratosis, a small number of mononuclear inflammatory cells infiltrated the dermis, and mild edema. In the samples treated with the exosomes it showed mild parakeratosis and the infiltration by a small number of mononuclear inflammatory cells and with mild edema. The photomicrograph of the treated group with loaded exosomes showed normal histological structure of dermis and epidermis.

**Fig. 12:**
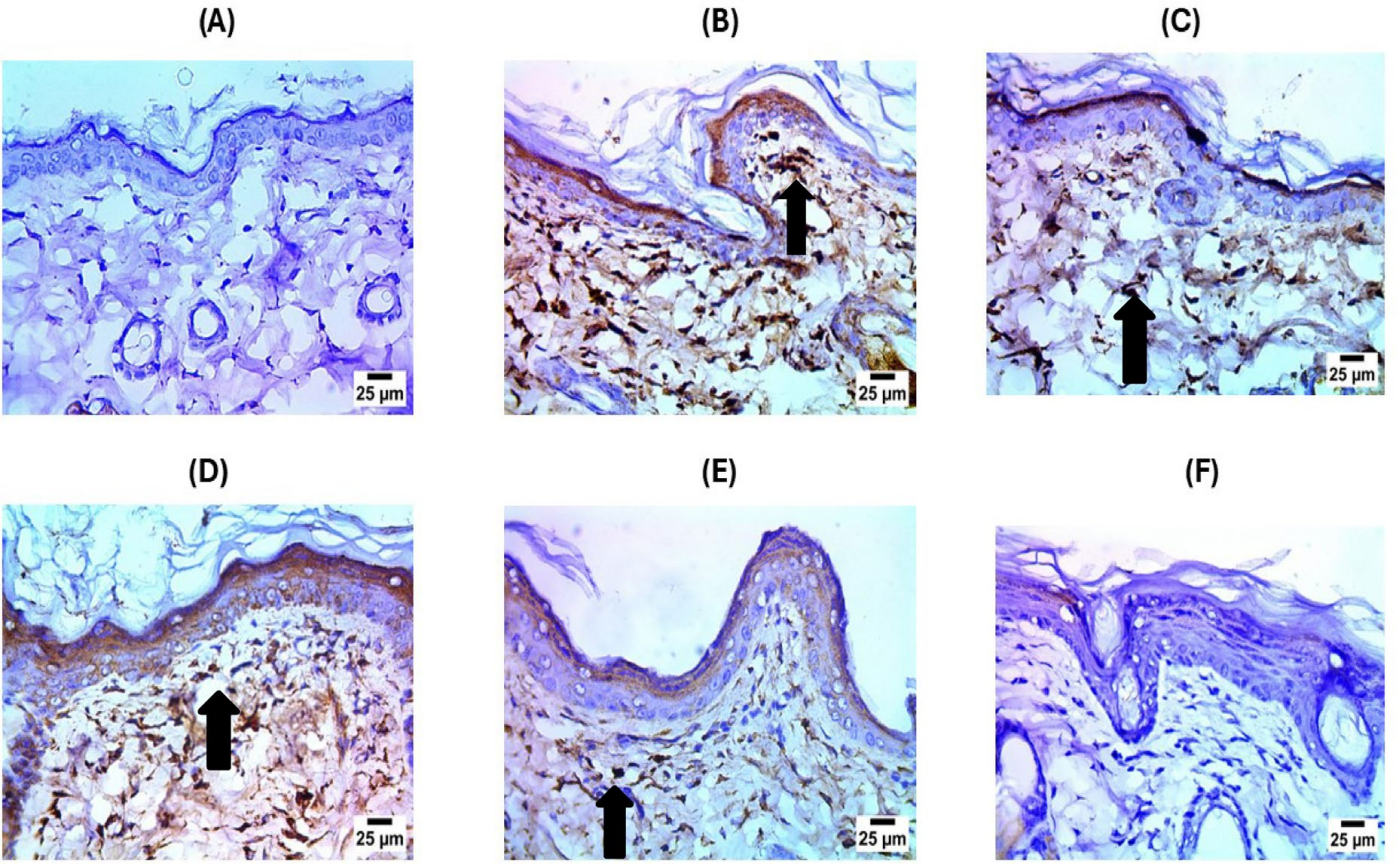
Photomicrographs of the immunohistochemistry results. This figure shows (**A**) negative expression of TGFβ in dermis in the negative control samples, (**B**) indicates sever positive expression of TGFβ (black arrow) in the untreated samples, (**C**) shows the treated group with Mesenchymal stem cells with moderate positive expression (black arrow) for TGFβ in dermis, (**D**) indicates the sample treated with EGCG Nanoparticles with severe positive expression for TGFβ (black arrow) in dermis, (**E**) indicates mild positive expression (black arrow) in the samples treated with exosomes and (**F**) indicates the sample treated with EGCG Nanoparticles loaded Exosomes with negative expression for TGFβ in dermis.

**Fig. 13:**
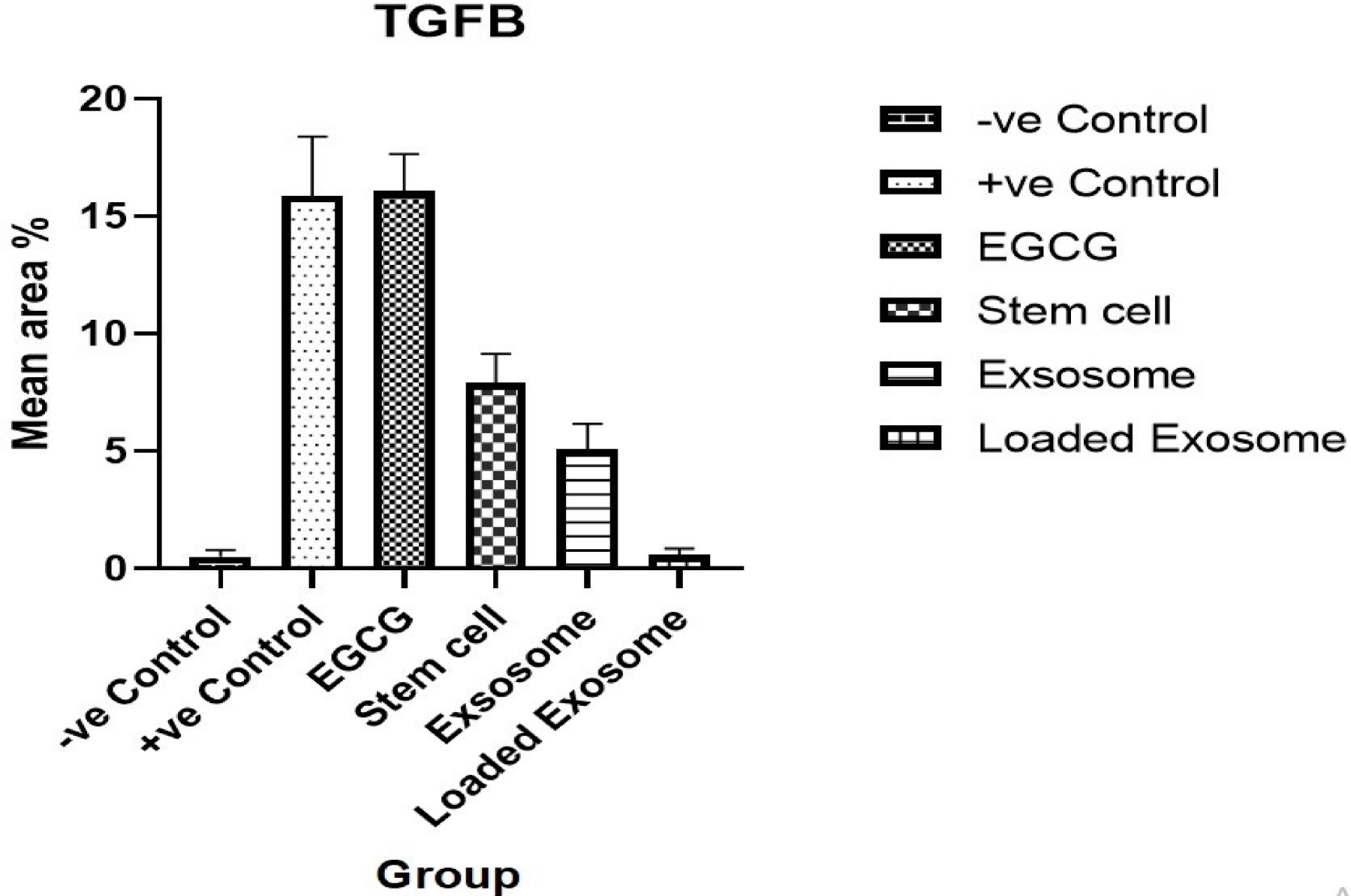
Showing Reaction area percent of TGFB and Data represented as a mean ± SD (*n* = 10), values indicate that these means of loaded exosome group were significantly variable than other group (*P* ≤ 0.0001) according to one-way ANOVA and Tukey tests.

The immunohistochemistry results showed that the control group had negative expression for TGFβ in the dermis, as shown in Figs. 12 and 13. Conversely, the Psoriasis induced group Photomicrograph showed severe positive expression for TGFβ in the dermis. Meanwhile, the treated group (MSCs) photomicrograph showed moderate positive expression for TGFβ in the dermis. While in EGN treatment, the expression of TGFβ dropped to a mild level. After the treatment with exosomes, the expression decreased to a mild expression of TGFβ. The photomicrograph of the treated group (EGN-Exo) had negative expression for TGFβ in the dermis.

## Discussion

Psoriasis is driven by a complex inflammatory network predominantly orchestrated by NF-κB, a key transcription factor that amplifies immune responses and impairs normal keratinocyte function. Its activation leads to the sustained production of pro-inflammatory cytokines, antimicrobial peptides, and cell survival proteins, all of which contribute to chronic inflammation^11^. In parallel, upregulation of the CDC25B pathway in psoriatic skin promotes abnormal keratinocyte proliferation through enhanced cell cycle progression. The inflammatory environment is further intensified by dysregulated T helper cell subsets, particularly Th17 and Th22, which secrete cytokines such as TNF-α, IL-17, and IL-22, perpetuating immune cell infiltration and tissue injury^12^. Additionally, an imbalance between pro-apoptotic Bax and anti-apoptotic Bcl-2 proteins impairs normal apoptosis, prolonging keratinocyte survival and contributing to the persistence of psoriatic lesions^13^as shown in Fig. 12.

Systemic therapy for psoriasis involves the use of biologics such as receptor fusion proteins and monoclonal antibodies, as well as small drugs like retinoids, cyclosporin, and methotrexate (MTX). However, biologics target inflammatory cytokines, while the majority of small molecules decrease immune activity or reduce cellular proliferation^14^.

Hepatotoxicity, nephrotoxicity, hypertension, tremors, hypomagnesemia, hyperkalemia, cancers^15^. Poor long-term patient compliance^16^ and anti-drug reactions^17^. These are among the harmful side effects that frequently affect the long-term use of these systemic medicines. Based on these side effects, scientists began to search for the main mechanism of psoriasis to provide alternative forms of treatment that are more effective and have fewer side effects. The finding of antimicrobial peptides (AMPs), which are excessively expressed in psoriatic skin and released through keratinocytes because of exposure to injury. β-defensins, S100 proteins, and LL37 are among the major researched psoriasis-triggering Amps. After being released through keratinocytes which were injured, LL37 linked or combines with self-genetic material from other injured cells to generate complexes. Toll-like receptor (TLR-9) in plasmacytoid dendritic cells (pDCs) are triggered by LL37 coupled to or linked to DNA. The activation of plasmacytoid dendritic cells (pDCs) serves an important role in promoting pathogenesis of psoriasis, specifically the generation of psoriatic plaques. This process is documented by the secretion of type I interferons (IFN-α and IFN-β) that result in phenotypic maturation of myeloid dendritic cells (mDCs)^14^.

Myeloid dendritic cells (mDCs) generate key pro-inflammatory cytokines as tumor necrosis factor (TNF)-α, interleukin (IL)−23 and IL-12, that result in activation the IL-22 and/or IL-23 signaling pathways. This activation initiate differentiation of Th22 and Th17 cell subsets, result in generation of a cascade of psoriasis-associated cytokines including interferon-gamma (IFN-γ), TNF-α, IL-22, and IL-17. These cytokines, in turn, exert their significant effects on keratinocytes, sustaining pathogenic cycle characteristic of psoriatic lesions and amplifying as well as triggering inflammatory responses^18^. Meanwhile, Th1 lymphocytes were commonly known by generation of interferon-gamma (IFN-γ) and tumor necrosis factor-alpha (TNF-α) which contribute to inflammatory milieu in psoriasis. Chemokines generated by keratinocytes as a result to these inflammatory mediators play a significant role in sustaining as well as triggering immune cell infiltration by facilitating the continuous recruitment of leukocytes among affected locations, which result in perpetuating the chronic inflammatory process^19^.

In addition to that, the AMP LL37 combines with RNA, initiating pDC activation with Toll-like receptor 7 (TLR7). Meanwhile, LL37–RNA complexes engage mDCs via TLR8, resulting in their activation as well as migration to draining lymph nodes. Once there, mDCs generate pro-inflammatory mediators as IL-12, tumor necrosis factor (TNF)-α, and IL-23, which further drive expansion and differentiation of Th1 as well as Th17 cell subsets, thereby perpetuating the characteristic inflammatory cascade of psoriasis^14^.

On the other hand, Interleukin-17 (IL-17) engages its receptor (IL-17R) on keratinocyte membrane, promoting intracellular signaling which results in activation of CARMA2 variants. Specifically, the CARMA2sh isoform is upregulated as a result of endoplasmic reticulum (ER) stress and IL-17 stimulation. CARMA2sh interacts with key adaptor proteins, including TRAF2, TRAF6, ULK2, RNF7, and DEPDC7, facilitating downstream signaling, which triggers activation of NF-κB. Through its interaction with MALT1 and BCL10, CARMA2sh triggers the CBM signaling complex, which initiates activation of the IKK complex (comprising IKKβ, IKKα, and IKKγ/NEMO). This activation is essential for NF-κB signaling, culminating in the translocation of NF-κB to the nucleus. Once inside the nucleus, NF-κB triggers transcription of genes encoding pro-inflammatory cytokines, keratins, chemokines, AMPs, and anti-apoptotic proteins, thereby sustaining inflammatory response among keratinocytes^20^as shown in Fig. 13.

**Fig. 14:**
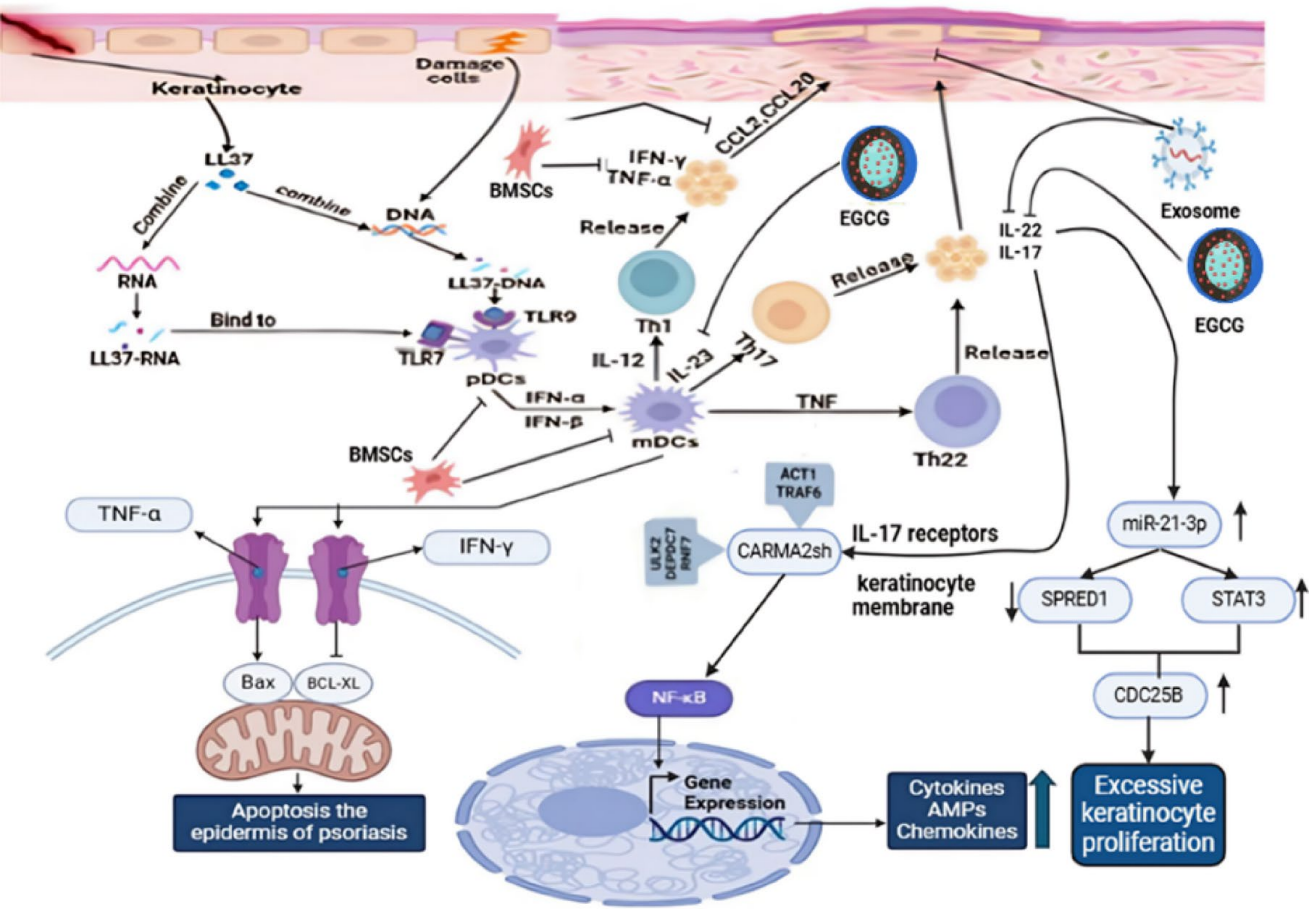
Mechanistic role of exosome-delivered EGCG in modulating psoriatic keratinocyte inflammation and apoptosis.

In psoriasis, TNF-α as well as IFN-γ modulate keratinocyte apoptosis by changing expression balance between Bax which is a pro-apoptotic protein, and Bcl-xL which is an anti-apoptotic regulator, result in initiating programmed cell death^13^. Abdallah et al. (2021) stated that “treatment of HaCaT cells with IL-22 induces a cell-signaling event favoring the activation of NF-κB and STAT3, which in turn enhances the transcription of the miR-21 gene.” This showed that IL-22 stimulation directly result in significantly upregulation of miR-21-3p, as evidenced by the finding that “expression levels of miR-21-3p increased over twofold (P < 0.05)^21^.

Li et al. (2020) further documented those consequences of this IL-22 driven upregulation miR-21-3p, stating that “coculture of NHEK with psoriatic DMSC induced a marked increase in expression levels of mRNA for MAPK3, CDC25B, and CDC25C, while decreasing expression levels of SPRED1 mRNA and protein in comparison with control DMSC treatment (P < 0.05 for all between cocultured with control and psoriatic DMSC).” This supports the role of IL-22 in driving NF-κB and STAT3 activation, leading to miR-21-3p expression, which subsequently results in SPRED1 suppression and a corresponding increase in CDC25B expression^22^.

The study on the modulation of PI3K/AKT signaling and density functional theory (DFT) modeling highlights the potential of selected pharmaceutical compounds in attenuating inflammation and oxidative stress in carrageenan-induced models, which can be related to the findings of our research on psoriasis^23^. In our investigation, the expression levels of interleukins IL-4 and IL-6, as well as apoptosis markers Bax and Bcl-2, were analyzed using ELISA techniques. The results demonstrated elevated levels of IL-4 and IL-6 in psoriasis-induced samples compared to control, indicating heightened inflammatory responses. Following treatments with Mesenchymal stem cells, EGCG nanoparticles, exosomes, and EGCG nanoparticle-loaded exosomes, the expression levels of these interleukins decreased, suggesting a reduction in inflammation. Similarly, the pro-apoptotic marker Bax was found to be upregulated in psoriasis samples, with levels increasing to 2.9 compared to the normal range of 1, while the anti-apoptotic marker Bcl-2 was downregulated to 0.3. Post-treatment observations revealed a decrease in Bax levels and an increase in Bcl-2 levels, indicating a shift towards reduced apoptosis and enhanced cell survival. These findings align with the broader implications of targeting inflammatory and apoptotic pathways, underscoring the therapeutic potential of modulating key molecular targets to mitigate disease pathogenesis and improve clinical outcomes.

MSCs have shown strong immunomodulatory effects through the suppression of pro-inflammatory cytokines and chemokines, such as TNF-α, IL-6, IL-17, CCL17, CCL20, and CCL27. A significant research study showed that MSCs can moderate the dysregulated Th1, Th2, and Th17 responses in psoriasis and prevent plasmacytoid dendritic cells (pDCs) from producing type I interferon (IFN-I)^24^and Zoheir et al. demonstrated that lipoic acid significantly reduces the levels of pro-inflammatory cytokines, thereby enhancing wound healing through its immunomodulatory and anti-inflammatory effects^25^. The ELISA results in our study support these findings, showing that MSC treatment significantly reduced IL-6 and IL-4 levels compared to the psoriasis-induced group (Fig. 7). Additionally, flow cytometry and apoptosis analysis (Fig. 10) revealed that MSCs partially restored the balance between pro-apoptotic (Bax) and anti-apoptotic (Bcl-2) markers, further confirming their regulatory role in inflammation and cell survival. On the other hand, exosomes derived from mesenchymal stem cells (MSC-Exo) have been shown to reduce proinflammatory cytokines like IL-17 and IL-23, as well as inhibiting the terminal complement activation complex C5b-9 in psoriatic skin. Topical application of MSC-Exo can alleviate IL-17 release by targeting neutrophil extracellular traps (NETs) in the stratum corneum, further mitigating inflammation^26^. Similarly, EGCG (epigallocatechin gallate) nanoparticles exhibited strong anti-inflammatory properties, as evidenced by reducing skin inflammation, decreasing infiltration of T cells in psoriatic lesions, lowering percentages of CD11c + dendritic cells (DCs) in splenic immunocytes as well as suppressing plasma levels of IL-17 A, IL-17 F, IL-22, IL-23, and malondialdehyde (MDA), indicating reduced oxidative stress and cytokine-driven inflammation^27^. Our experimental data aligns with these mechanisms, demonstrating that EGCG nanoparticles (EGN) lowered IL-6 and IL-4 expression (Fig. 7), reduced NF-κB and CDC25B pathway activation (Fig. 8), and improved histopathological features (Fig. 11) by minimizing parakeratosis and inflammatory cell infiltration.

The present study elucidates the molecular dynamics of psoriasis through the analysis of ELISA results, which revealed elevated expression levels of the CDC25B and NF-kB pathways in affected samples, with values reaching 3.4 and 2.1, respectively. These findings indicate a significant upregulation compared to the baseline level of 1, suggesting a pivotal role of these pathways in the pathogenesis of psoriasis. Post-treatment observations demonstrated a marked reduction in these expression levels, approaching normal ranges, thereby indicating the potential efficacy of the therapeutic interventions in modulating these pathways and mitigating disease symptoms. In contrast, existing literature, such as the study on Viramidine-loaded aptamer-nanoparticles, illustrates a distinct therapeutic paradigm targeting the CDC25A/p53/PI3k pathway to induce cell cycle arrest in hepatoma cells^28^. This juxtaposition underscores the specificity of molecular targets in different disease contexts, with our research highlighting the therapeutic potential of modulating CDC25B and NF-kB in psoriasis management. Collectively, these insights contribute to the broader understanding of targeted molecular therapies, emphasizing the importance of tailored interventions in addressing complex diseases.

CD105 is a key positive marker for MSCs and is required for their multilineage differentiation potential, especially toward osteogenic and chondrogenic lineages. CD73 is another canonical positive marker for MSCs, associated with their immunomodulatory properties and multipotency. CD34 is a well-established marker for HSCs and endothelial progenitor cells. Its absence is used to confirm MSC identity, as MSCs are typically CD34-negative. MSCs are characteristically CD14-negative, which helps distinguish them from monocyte/macrophage lineage cells in culture^29^.

The results of characterization of MSCs by flow cytometry showed well-characterized MSCs as the two positive CD markers (CD 105, CD 73) appeared in high expression levels, and the two negative CD markers (CD34, CD14) appeared in low expression levels. These results were in agreement with the results obtained by^30^. CD9 and CD63 are tetraspanins, integral membrane proteins that are commonly enriched on the surface of exosomes. These proteins are involved in exosome formation and cargo sorting and serve as classical markers for small EVs of endosomal origin. The MISEV2018 guidelines recommend that at least one transmembrane protein (e.g., CD9, CD63, or CD81) be used for EV characterization to confirm their membrane origin and integrity. HSP70 (Heat Shock Protein 70) is a cytosolic chaperone protein frequently found in the lumen of exosomes. It participates in protein folding and cellular stress responses and is often used as an internal (luminal) marker of exosomes. MISEV2018 categorizes HSP70 as a representative cytosolic protein commonly associated with EVs, suggesting its inclusion for robust characterization^31^.

The results of characterization of MSCs-derived exosomes by flow cytometry showed that exosomes were characterized successfully as the tested markers (HSP 70, CD 9, and CD 63) appeared in high expression levels. These results were in agreement with the results obtained by^32^.

The results of transmission electron microscopy imaging showed a clear indication of the encapsulation of EGCG with nanoparticles, and these nanoparticles with exosomes. The presence of different sizes of the nanoparticles with a diameter of 13.5 nm for EGCG nanoparticles, 73.66 nm for exosomes and 96.54 nm for the loaded exosomes confirms the versatility of these nanoparticles and also the increase in size of the loaded exosomes compared with the exosomes indicates the proper encapsulation of EGCG nanoparticles in the exosomes and variation in size between them indicates that the combination of the EGCG nanoparticles changes the structural features. The results of the PDI of EGCG nanoparticles, Exosomes, and loaded exosomes confirmed the successful distribution, stability, and homogeneity of these nanoparticles. And the results of the entrapment efficiency showed the successful nanoparticle encapsulation of EGCG and the effective loading of EGCG nanoparticles in the exosomes. These results were in agreement with the results obtained by^33^.

The results of in vitro release of EGCG showed that EGCG nanoparticles manifested a slow release of 99.5% over 48 h, and the loaded exosomes appeared with 82.1% release over 48 h and this slow release demonstrates the stability of these nanoparticles and their potential therapeutic efficiency These results were in agreement with the results obtained by^34^.

The applied treatments (MSCs, MSCs-derived exosomes, EGCG nanoparticles, and EGCG nanoparticles-loaded exosomes) showed significant effects in treating the rats induced with psoriasis as compared with the induced rats that were left without any treatment (positive control). The results of ELISA showed the expression levels of interleukins-4, and IL-6 and it showed that psoriasis highly increases the expression of the both interleukins and that psoriasis promotes the inflammatory effects and then demonstrated that the applying treatment can reduce these inflammatory effect by decreasing the expression levels of the interleukins-4, and IL-6.where, the EGCG nanoparticles- loaded exosomes showed the best anti-inflammatory effects as compared with the other treatments These results were in agreement with the results obtained by^35^.

The results of flow cytometric analysis demonstrated dysregulated apoptosis in psoriasis, which is distinguished by increased early and late apoptotic cell populations. The treatments, notably samples with EGN-Exo, show great potential for restoring cellular homeostasis by significantly reducing apoptosis and necrosis. Treatment groups with MSCs, EGN, and Exo all show lower Bax expression than the Ps group, indicating that these therapies are used to regulate apoptosis. Among these, the treated sample with the EGN-Exo group has the highest reduction, bringing Bax expression closer to normal. The samples treated with MSCs, EGN, and Exo treatment groups all partially restore Bcl-2 expression, though to varying degrees. The treated sample with the EGN-Exo group exhibits the highest efficacy, comparable to the control group. These results were in agreement with the results obtained by^36^.

In the ELISA analysis, the treatments of the samples with MSCs, EGN, Exo, and EGN-Exo seem to attenuate this upregulation to varying degrees. Notably, the treated samples with MSCs and EGN treatment groups showed only minor reductions in CDC25B expression, but the treated samples with Exo and EGN-Exo treatments generated a more significant decrease, bringing expression closer to normal levels. The sample treated with the EGN-Exo group showed the greatest reduction in CDC25B expression, demonstrating that the combination of EGN and exosomes is extremely effective. Treatment with MSCs EGN and Exo decreased NF-κB expression compared to the Ps group, indicating anti-inflammatory advantages. These results were in agreement with the results obtained by^37^. Samples treated with EGN-Exo exhibit the highest reduction, so it has the best effect.

In the CBC analysis, samples treated with MSCs, EGN, Exo, and EGN-Exo showed a difference in the leukocyte count reduction, which clarified their immunomodulatory and anti-inflammatory effects. These results were in agreement with the results obtained by^38^. The increased T helper cells in the psoriasis sample is consistent with immunological activation observed in the inflammatory disease. The elevated T helper cells are linked to the illness with excessive immune response^39^. EGN-Exo is a promising treatment as the T helper cells were greatly reduced, but there is no significant difference between the other treatments. The level of the elevated platelets in the psoriasis sample is consistent with systemic inflammation observed in autoimmune illness. These results were in agreement with the results obtained by^38^. The most significant effect was the treated sample with EGN-Exo. Elevated MPV in the psoriasis sample indicates platelet activation, which is common in inflammatory states. The greatest effect was for the treated sample with EGN-Exo.

The results of histopathology showed the usual histological structure of dermis and epidermis in the negative control. While in the sample, which is untreated, Parakeratosis and severe edema were indicated, the treated group, which was injected with mesenchymal stem cells, outcomes confirm normality of histological structure for both epidermis and dermis as well as the presence of parakeratosis. The tissue’s normal composition was primarily restored^40^. In the sample, which was treated with EGN, it was discovered that these properties were significantly diminished, indicating that the normal structure of the skin was restored and the inflammatory process was successfully suppressed^41^. While in the sample treated with Exosomes, a mild parakeratosis and a reduction in the edema and the infiltration of the mononuclear inflammatory cells were observed after the treatment with MSCs-derived exosomes^42^. The treated group with EGN loaded Exosomes displays a histological similarity to the healthy negative control group (CT) with restoration of normal skin architecture, this suggests that the EGN-Exo may have a therapeutic effect in alleviating the symptoms of psoriasis, evidenced by reduced inflammation and normalization of the skin layers These results were in agreement with the results obtained by^7^. From a regenerative medicine standpoint, these outcomes align with prior studies supporting the role of **mesenchymal stem cells (MSCs)** as well as their derivatives as **exosomes**, in triggering tissue repair, decreasing inflammation, and developing regeneration of skin. For instance, topical applications of MSC-derived exosomes have shown efficacy in alleviating imiquimod-induced psoriatic inflammation and improving dermal architecture^43,44^.

The results of immunohistochemistry showed that the control group dermal TGF-β expression was negative, which is typical for normal healthy tissue. Conversely, the psoriasis-induced group indicates significantly positive expression of TGF-β in the dermis. The excessive expression of TGF-β was mainly due to tissue damage, triggering repair pathways. Finally, the treated group with mesenchymal stem cells outcomes confirm positive mild expression of TGF-β in dermis, which indicates the capability of mesenchymal stem cells to repair tissue damage of tissues as well as promote homeostasis of tissues^45^. IHC confirmed that EGCG nanoparticle therapy significantly reduced TGFβ expression. This suggests that by changing large inflammatory pathways, nanoparticles help solve the disease^46^.

The expression levels of TGFβ decreased after the treatment with MSCs-derived exosomes, and this can indicate the role of exosomes in the regulation of the cytokine, and that exosomes can not only reduce the inflammation but can also enhance tissue repair and help to restore the normal dermal structure^47^. The treated group with EGN-loaded Exosomes exhibited negative expressions for TGF-β in the dermis, like the negative control. This result suggests that treatment with EGN-Exo may effectively inhibit TGF-β expression, which helps in restoring normal keratinocyte differentiation and reducing inflammation, ultimately leading to improved clinical outcomes. These results agreed with the results obtained by López González et al. (2017).

## Materials and methods

### Material

The following materials and equipment were used in this study: Imiquimod cream (Aldara 5%, MEDA, Sweden); EGCG nanoparticles, dialysis membrane (Sigma-Aldrich, USA); DMEM media, phosphate-buffered saline (PBS), trypsin (Thermo Fisher, USA); ELISA kit (Elabscience, USA); UV-BioTek microplate reader (BioTek Instruments, USA); transmission electron microscope (JEOL Ltd., Japan); and dynamic light scattering analyzer (Malvern Panalytical, USA).

### Study design

The experimental design has included 6 groups of five male Wistar rats (Purchased from and housed at the Animal House of National Research Centre, Egypt) aging between 8 and 12 weeks ta(average 150–200 gm) categorized into a Negative control (healthy group) (CT), Positive control (induced with psoriasis and untreated group) (Ps), Four Positive control (induced with psoriasis and treated group) (MSCs), (Exo), (EGN) and (EGN-Exo).

### Isolation and characterization of stem cells (morphology and flow)

Wistar rats that were 6–8 weeks old and were euthanized via cervical dislocation after deep anesthesia achieved by (xylazine/Ketamine mixture) then had their bone marrow extracted. After rinsing the skeletons in 70% ethanol, the skin was cut off around the rear limbs and pulled toward the foot to avoid fur contamination^48^. The hind limbs were dissected from the trunk along the spinal cord, being careful not to injure the femur, after the foot was removed at the ankle bone. Until further processing, the dissected limbs were kept on ice in DMEM supplemented with one x penicillin/streptomycin. Each hind leg was cut in half at the knee joint while maintaining sterility. The tibia and femur were then stripped of muscle and connective tissue by scraping the diaphysis and removing tissue from the ends of the bones. DMEM with 1x penicillin/streptomycin was used to keep cleaned bones on ice. Using a rongeur, the extremities of the tibia and femur were sliced below the marrow cavity in order to extract bone marrow. The marrow plugs were collected into 10-ml tubes on ice after being flushed from the bone ends using a 27-gauge needle connected to a 10-ml syringe filled with complete media. Trypan blue exclusion and hemocytometer counting were used to evaluate the cell viability after the cell solution was filtered through a 70-µm mesh to eliminate debris. In a humidified chamber, bone marrow cells were cultivated in 95-mm culture dishes at a density of 25 × 10,625 \times 10^6 cells/ml in 1 ml of complete media. The cells were then incubated at 37 °C with 5% CO_2_^49^.

Nonadherent cells were removed after 3 h by changing the medium, which was replaced with fresh complete medium. After an additional 8 h, the medium was replaced again and subsequently every 8 h for up to 72 h. Adherent cells (passage 0) were washed with phosphate-buffered saline (PBS), and fresh medium was added every 3–4 days. Spindle-shaped adherent cells were observed as individual cells by day 3 under phase-contrast microscopy, forming colonies by days 4–8. Within two weeks, cultures had attained 65–70% confluence, showing fibroblastic colonies with a few hematopoietic cells scattered throughout. Following two weeks, cells were removed using 0.5 ml of 0.25% trypsin for two minutes at room temperature after being cleaned with PBS. Cells were moved to a 25 cm^2^ flask for further culture after trypsin was neutralized with 1.5 ml of complete media. Confluence was usually reached after seven days, with the medium being replaced every three days with six milliliters of fresh medium^50^.

The identification and characterization of stem cells, particularly **mesenchymal stem cells (MSCs)**, are heavily reliant on the expression or absence of specific **cluster of differentiation (CD)** surface markers. Among these, **CD105**, **CD73**, **CD34**, and **CD14** are commonly used to define stem cell populations based on guidelines from the International Society for Cellular Therapy (ISCT) and other peer-reviewed sources^51^.

Meanwhile, Inverted microscopy was utilized to locate mesenchymal stem cells (MSCs) depending on their spindle-shaped morphology as well as adhesion to the tissue culture flask. The surface markers of cultivated mesenchymal stem cells (CD90, CD105, CD73 and CD14) were exposed to flow cytometric analysis. After being washed, 50,000 cells were re-suspended in (PBS) phosphate buffer saline which were supplied with 3% of (FBS) fetal bovine serum diluted to saturating concentrations with ratio (1:100). Antibodies against CD90, CD105, CD73 and CD14 (anti-CD) conjugated with cyanine 5 (CY5), allophycocyanin (APC), fluorescein isothiocyanate (FITC) dyes, phycoerythrin (PE), correspondingly, were incubated at normal room temperature for 45 min in 300 µL of cell suspension. Flow cytometry was generated. After that, forward scatter analysis was utilized to evaluate samples^52^.

### Isolation and characterization of stem cells’ exosomes

MSCs in passage IV (5 × 106 cells/mL) were used. The MSCs were treated with 0.5% BSA and cultivated in RPMI medium devoid of FBS. To get rid of debris, the cell-free supernatant was collected and centrifuged for 20 min at 2000xg. A Beckman Coulter Optima L 90 K ultracentrifuge was then used to further centrifuge the resultant supernatant at 100,000×g for one hour at 4 °C. The purpose of this phase was to separate the exosomes from the other parts of the cell. A second round of ultracentrifugation was performed under the same circumstances after the cells had been rinsed in serum-free media with 199 HEPES 25 mM. The goal of this procedure was to guarantee the exosomes’ purity^50^. Using isothiocyanate-conjugated anti-human monoclonal antibodies, such as anti-CD63, anti-CD9, and anti-HSP70, flow cytometry was used for characterization. Exosomes were extracted, cleaned, and then resuspended in PBS containing 3% FBS. After that, the samples were tested using a spray assay^50^. TEM, which was connected to a CCD camera at an accelerating voltage of 200 kV, was used to analyze the size and shape of exosomes^53^. A thin layer of amorphous carbon was placed on a copper grid (300 mesh), and one drop of the exosomes was placed on it. After allowing the solution to air dry, the sample was inspected under a microscope or measured for nanoparticles using micrographs. The sample is thoroughly examined again at 25 °C, pH 3–4, and a scattering angle of 90° to conduct dynamic light scattering (DLS) for further characterization^54^.

### Preparation and characterization of EGCG nano

Using the ionic gelation technique, EGCG nanoparticles (EGN) were created by adding a dropwise solution of tripolyphosphate (TPP) (0.1%, w/v; pH 3) to a chitosan solution (0.1% w/v in 0.175% v/v acetic acid; pH 3) that contained EGCG (0.05% w/v) for 10 min at room temperature (25 °C) while being magnetically stirred^55^. The EGN solution was then dialyzed to remove any impurities using a dialysis membrane^56^. TEM, which was connected to a CCD camera at an accelerating voltage of 200 kV, was used to analyze the size and shape of EGN. On a thin layer of amorphous carbon that had been deposited on a copper grid (300 mesh), one drop of the EGN was placed^57^. Following air drying, the sample was inspected under a microscope or using micrographs to quantify the nanoparticles. Characterization is carried out utilizing DLS for further assurance, and NPs were thoroughly analyzed at 25 °C, pH 3–4, and a scattering angle of 90°^58^.

### Loading of EGN into exosomes and its release

Epigallocatechin gallate (EGCG) was loaded into exosomes using a high-concentration incubation method with vigorous shaking. Purified exosomes were incubated with a high concentration of EGCG at room temperature for 2 h under continuous mild shaking to facilitate passive diffusion of EGCG across the exosome membrane^59^.

TEM, which was connected to a CCD camera at an accelerating voltage of 200 kV, was used to analyze the size and shape of EGN. On a thin layer of amorphous carbon that had been deposited on a copper grid (300 mesh), one drop of the EGN was placed. After allowing the solution to air dry, the sample was inspected under a microscope or measured for nanoparticles using micrographs. Characterization is carried out utilizing DLS for further assurance, and the NPs were thoroughly inspected at 25 °C, pH 3–4, and a scattering angle of 90°. The redispersed NPs were dissolved in a 1% v/v solution for 30 min after being mixed with an acetic acid solution to determine the entrapment efficiency. To isolate the EGCG, the whole solution was centrifuged for 15 min at a velocity of around 5000 × g through a Millipore centrifugal filter with a 100-kDa cutoff. EGN-Exo was loaded onto a dialysis membrane to test the drug’s in vitro release^9^. The phosphate-buffered fluid (pH 7.4) was used to simulate the physiological milieu of the body by submerging each dialysis membrane. Every vial was put in a shaker that rotated at 150 rpm and had a temperature of 37 °C. The buffer was collected and replaced with a fresh buffer at selected time points: 0, 4, 8, 12, 24, and 48 h. The concentration of EGCG in the collected solutions was determined at 278 nm.

### Induction of psoriasis

In the investigation, thirty male Wistar rats weighing an average of 150–200 g and aged between 8 and 12 weeks were employed. The National Research Center’s (NRC) Animal Housing Department is where the rats were grown and kept. The animals received treatment in a climate-controlled space with 12-hour cycles of light and dark. They were fed regular food and drink and kept in plastic cages covered with mesh wire. Before any interference, the animals were left for a week to acclimate to their new surroundings. The use of animals was authorized by the National Research Center’s (NRC) Medical Research Ethics Committee (MREC) and carried out in compliance with the rules for the care and use of laboratory animals. The ARRIVE standards were followed in reporting this investigation, and the National Institutes of Health’s (NIH) guidelines for the care and use of animals in experiments were followed in handling and conducting the experiments. To begin the psoriasis induction process, each rat’s back hair was shaved in an area around the size of a coin (3 cm in diameter) to reveal the skin at the cervical region of the spine, which covers the seven vertebrae right below the head. For seven days in a row, 1/3 of a sachet of imiquimod cream was administered every day after the skin was exposed. This protocol was used for the untreated and treated positive control groups^60^.

### Injection of treatment

Rats were divided into six groups for the injection: treatment groups that received MSCs, exosomes, EGN, and EGN-Exo; negative control; and positive control. Each rat received an injection of 0.1 ml of the therapeutic drug for every 1 cm² of diseased skin. The region right beneath the psoriatic lesion was the focus of subepidermal injections. The treated area was left undisturbed for one week to allow the therapeutic effects to manifest.

### Biochemical analysis

#### ELISA assay

##### Expression levels of IL-4, IL-6 proteins, as well as apoptosis markers, include Bax and Bcl-2

After identifying the wells for the diluted blank, standard, and sample by the manufacturer’s instructions, 100 µL of each dilution was added to the corresponding wells. The plate was covered with the supplied sealer and then incubated at 37 °C for 90 min. The liquid was removed after incubation, and each well immediately received 100µL of the Biotinylated Detection Antibody working solution. After thoroughly covering the plate with a fresh sealer, it was incubated at 37 °C for an hour. Following the removal of the liquid from each well, 350 µL of wash buffer was added to each well. The wells were aspirated or decanted after a minute of soaking, and new absorbent paper was used to pat dry the plate. Three iterations of this washing process were performed. After filling each well with 100 µL of HRP Conjugate working solution, the plate was sealed with fresh sealer and incubated at 37 °C for 30 min^61^. The washing process was repeated five times, after which the liquid was removed again and incubated. After 90 µL of substrate reagent was added to each well, the plate was incubated for 15 min at 37 °C without being exposed to light. Following the incubation period, 50 µL of stop solution was added to each well to block the process. Lastly, a microplate reader was used to instantly calculate the optical density (OD) values for wells at a wavelength of 450 nm. For example, biotinylated anti-IL-4 antibody (E-EL-R0014) was used to detect IL-4, biotinylated anti-IL-6 antibody (E-EL-R0015) was used to detect IL-6, biotinylated anti-Bax antibody (E-EL-R0098) was used to detect pro-apoptotic marker Bax, and anti-apoptotic marker Bcl-2 was detected by anti-Bcl-2 monoclonal antibody (E-EL-R0096).

##### Expression levels CDC25 & NF-kB markers

Based on the Elabscience Rat NF-kB/CDC25 ELISA Kit lab handbook. Following the determination of the wells for the diluted standard, blank, and sample, 100 µL of each dilution was added to the corresponding wells. After applying the included sealer, the plate was incubated for ninety minutes at 37 °C. Following incubation, 100 µL of the Biotinylated Detection Antibody working solution was promptly added to each well, and the liquid was disposed away. After applying a fresh sealer, the plate was incubated for an hour at 37 °C. Following the decantation of the liquid from each well, 350 µL of wash buffer was added to each well. After soaking the wells for a minute, the buffer was decanted or aspirated, and the plate was patted dry with fresh absorbent paper. Three times, this washing procedure was carried out. Following the addition of 100 µL of HRP Conjugate working solution to each well, the plate was sealed with a fresh sealer and incubated for 30 min at 37 °C. After incubation, the liquid was once more decanted, and the previously mentioned washing procedure was carried out five times. After adding 90 µL of substrate reagent to each well, the plate was incubated for about 15 min at 37 °C without being exposed to light. 50 µL of stop solution was applied to each well to halt the reaction after the incubation time. Lastly, a microplate reader was used to measure the optical density (OD) values of the wells instantly at a wavelength of 450 nm.

#### Complete blood count (CBC)

The rat’s blood was collected intracardially for CBC employing “Indexx LaserCyte” laser flow cytometry analyzer immediately before euthanasia under anesthesia (Xylazine/Ketamine Mixture). Microscopic inspection of poikilocytosis blood corpuscles was performed on a blood smear previously obtained and stained utilizing Giemsa’s method. Poikilocytosis was characterized semi-quantitatively according to comparable studies, employing the following criteria (non-existent (0%), rare (0.05 to 0.5%), moderate (< 0.5%), modest (< 3 to 10%). Number as well as type of poikilocytosis was documented as proportions of red blood cells. Relying on semi-quantitative examination of degenerative disorders, white blood cells were categorized for each percentage as follows: few (5–10%), mild (11–30%), numerous (> 30%). Utilizing a miotic type 102 M binocular light microscope with magnification of 900 times, 1000 white blood cells, and 2000 erythrocytes were examined as well as counted within the original stained smear^62^.

#### Flow cytometry

##### Apoptosis profiling

FACS Lysing Solution was developed to lyse red blood cells for utilization in flow cytometric applications. Cell apoptosis was detected using flow cytometry, the FITC Annexin V kit, and propidium iodide (Ann V-PI). Cells were gently mixed with 5 µl of propidium iodide solution and 5 µl of FITC Annexin V and then incubated for 15 min at room temperature to avoid light exposure. The following cells were stained by kit’s instructions, they were given binding buffer and analyzed by a flow cytometer utilizing at 488 nm as the excitation^28,58,63^.

#### Pathological assay

##### Hematoxylin & Eosin staining (Histopathology staining)

10% of neutral buffer formalin was utilized to fix the specimens of skin that were taken from the rats, followed by trimming, washing in water, dehydrating in ascending ethyl alcohol grades, laying out in xylene, and embedding in paraffin. After processing, thin Sects. (4–6 µ) were stained utilizing Hematoxylin & Eosin stain^64^.

##### Immunohistochemistry staining protocol

Avidin-biotin-peroxidase complex (ABC) technique was utilized to mount sections of paraffin. Chemicals required to perform the Avidin biotin-peroxidase complex were introduced after sections from each group were incubated with those antibodies. Marker expression was tagged with peroxidase and dyed with diaminobenzidine to identify the antibody-antigen complex. Secondary or primary antibodies were substituted with non-immune serum as negative controls. IHC-stained sections were examined using an Olympus BX-63 microscope. Calculations of immunohistochemistry results by identifying reaction area percentage in 10 microscopic fields utilizing Image J 1.53t^65^.

### Statistical analysis

The data obtained in the present work were represented in figures and tables as the mean *±* standard error (SE). All assays were repeated three times (*n* = 3). A one-way ANOVA analysis (GraphPad Prism, version 8.0.2) was used. **a**: P values < 0.01 were considered highly statistically significant when comparing the positive control (Ps) with the control, **b**: P values < 0.05 were considered statistically significant when comparing the treated groups (Ps + SCs, Ps + EGN, and Ps + EXO) with the positive control. **c**: P values < 0.01 were considered highly statistically significant when comparing the treated group (Ps + EGN-EXO) with positive control.

## Conclusion

This study emphasizes how mesenchymal stem cells (MSCs), exosomes produced from MSCs, and nanotechnology-based procedures can be utilized for managing psoriasis, a complicated chronic inflammatory condition. The strongest evidence of therapeutic efficacy among the assessed treatments was shown by exosomes loaded with EGCG nanoparticles. Suppression of important inflammatory pathways (CDC25B and NF-kB) enhanced apoptotic regulation (normalization of Bax and Bcl-2), and decreased pro-inflammatory cytokines (IL-4 and IL-6) supported these findings. The restoration of normal leukocyte numbers, T-helper cell activity, and platelet activation markers (PLT and MPV) further demonstrated how well this treatment regulated.

## Limitations

Despite these promising findings, the study has several limitations. The use of an animal model, while widely accepted, as expected, may not fully replicate the complexity of human psoriasis. Furthermore, although being well monitored and regulated, the results’ statistical power is limited by the relatively small sample size, and the emphasis on immediate results ignores the therapies’ long-term safety and effectiveness because there isn’t yet enough time to ascertain this element. Future research should prioritize clinical trials to validate these findings in human subjects, explore the undeniable potential synergy of EGN-Exo with existing therapies, and investigate the molecular mechanisms underlying its dual action on CDC25B and NF-κB.

## Electronic supplementary material

Below is the link to the electronic supplementary material.

Supplementary Material 1

## Data Availability

All data are available upon request from the corresponding author.

## Abbreviations

ABC: Avidin-Biotin-Peroxidase Complex
AMPs: Antimicrobial Peptides
APC: Allophycocyanin
CBC: Complete Blood Count
CDC25: Cell Division Cycle 25
Ct: Control Group
CY5: Cyanine 5
DCs: Dendritic Cells
DLS: Dynamic Light Scattering
DMEM: Dulbecco’s Modified Eagle Medium
EE: Entrapment Efficiency
EGCG: Epigallocatechin Gallate
EGN: EGCG Nanoparticles
ELISA: Enzyme-Linked Immunosorbent Assay
ER: Endoplasmic Reticulum
EXO: Exosomes
FITC: Fluorescein Isothiocyanate
IFN-α and IFN-β: Interferons Alpha and Beta
IFN-γ: Interferon Gamma
IHC: Immunohistochemistry
IL-4,6: Interleukins 4,6
MDA: Malondialdehyde
mDCs: Myeloid Dendritic Cells
MREC: Medical Research Ethics Committee
MSC: Mesenchymal Stem Cells
MTX: Methotrexate
NETs: Neutrophil Extracellular Traps
NF-κB: Nuclear Factor Kappa B
NRC: National Research Center
OD: Optical Density
PBS: Phosphate-Buffered Saline
pDCs: Plasmacytoid Dendritic Cells
PDI: Polydispersity Index
PE: Phycoerythrin
PI: Propidium Iodide
Ps: Psoriasis-Induced
SE: Standard Error
TEM: Transmission Electron Microscopy
TGFβ: Transforming Growth Factor-Beta
TH cells: T-Helper Cells
TLR: Toll-Like Receptors
TNF-α: Tumor Necrosis Factor Alpha
TPP: Tripolyphosphate
WHO: World Health Organization
