# Supplementary material for: Exosome mediated delivery of Epigallocatechin 3 gallate as a novel approach to alleviate psoriasis symptoms through cytokine and apoptotic pathway modulation
Source: Sci Rep. 2025 Aug 16;15:30013. doi: 10.1038/s41598-025-10886-2 (PMC12357947; doi:10.1038/s41598-025-10886-2)
Supplement: Supplementary file 1 — Supplementary Material 1 [file 41598_2025_10886_MOESM1_ESM.docx]

# **8. Supplementary data**

Supplementary Figure S1: The complete gating strategy for the flow cytometry. The unstained, single-stained, and double-stained samples were investigated.
